# Kupffer cells induce Notch-mediated hepatocyte conversion in a common mouse model of intrahepatic cholangiocarcinoma

**DOI:** 10.1038/srep34691

**Published:** 2016-10-04

**Authors:** Maiko Terada, Kenichi Horisawa, Shizuka Miura, Yasuo Takashima, Yasuyuki Ohkawa, Sayaka Sekiya, Kanae Matsuda-Ito, Atsushi Suzuki

**Affiliations:** 1 Division of Organogenesis and Regeneration, Medical Institute of Bioregulation, Kyushu University, 3-1-1 Maidashi, Higashi-ku, Fukuoka 812-8582, Japan; 2 Division of Transcriptomics, Medical Institute of Bioregulation, Kyushu University, 3-1-1 Maidashi, Higashi-ku, Fukuoka 812-8582, Japan; 3 Core Research for Evolutional Science and Technology, The Japan Agency for Medical Research and Development, 1-7-1 Otemachi, Chiyoda-ku, Tokyo 100-0004, Japan

## Abstract

Intrahepatic cholangiocarcinoma (ICC) is a malignant epithelial neoplasm composed of cells resembling cholangiocytes that line the intrahepatic bile ducts in portal areas of the hepatic lobule. Although ICC has been defined as a tumor arising from cholangiocyte transformation, recent evidence from genetic lineage-tracing experiments has indicated that hepatocytes can be a cellular origin of ICC by directly changing their fate to that of biliary lineage cells. Notch signaling has been identified as an essential factor for hepatocyte conversion into biliary lineage cells at the onset of ICC. However, the mechanisms underlying Notch signal activation in hepatocytes remain unclear. Here, using a mouse model of ICC, we found that hepatic macrophages called Kupffer cells transiently congregate around the central veins in the liver and express the Notch ligand Jagged-1 coincident with Notch activation in pericentral hepatocytes. Depletion of Kupffer cells prevents the Notch-mediated cell-fate conversion of hepatocytes to biliary lineage cells, inducing hepatocyte apoptosis and increasing mortality in mice. These findings will be useful for uncovering the pathogenic mechanism of ICC and developing prevenient and therapeutic strategies for this refractory disease.

Intrahepatic cholangiocarcinoma (ICC), the second most pervasive primary malignancy in the liver, is accompanied by delays in diagnosis and a poor prognosis, and its incidence and mortality rates are increasing worldwide^1^,^2^. Although ICC is thought to be a tumor arising from malignant transformation of cholangiocytes composing the intrahepatic bile ducts^3^,^4^, it has also been suggested that ICC can arise from transformed hepatocytes, because patients with viral hepatitis often develop ICC^5^,^6^,^7^. In our previous study, we conducted lineage-tracing analyses on hepatocytes labeled with heritable, cell type-specific reporters, and found that ICC can be generated by biliary lineage cells derived from hepatocytes in a mouse model of ICC induced by administration of thioacetamide (TAA)^8^. These findings may help to explain why patients with viral hepatitis often develop ICC. In these patients, a part of the hepatocytes infected with hepatitis viruses may be converted into biliary lineage cells and become a source of ICC.

Similar to the case for the mouse TAA model of ICC, cell-fate conversion can be observed in many types of injured/diseased tissues and organs in mammals. For example, acinar cells can change their fate to that of hepatocyte-like cells in the damaged pancreas of adult rats maintained on a copper-deficient diet^9^, and human gastric epithelial cells can be converted into cells that form intestinal villi during intestinal metaplasia induced by mucosal injury associated with *Helicobacter pylori* infection^10^,^11^. In mouse models of diabetes, β cells constituting islets of the pancreas dedifferentiate into progenitor-like cells, resulting in loss of insulin secretion from β cells^12^. Moreover, mouse Schwann cells infected with the leprosy bacterium can acquire the properties of progenitor-like cells, suggesting a correlation between cellular reprogramming and host–pathogen interactions^13^. Thus, it is suggested that the cell-fate conversion caused by damage to cells is closely related to the onset and progression of various diseases. Clarification of the mechanisms underlying these injury-induced types of cellular reprogramming will be important for the development of new medical technologies toward disease prevention and therapy. However, such mechanisms remain largely unknown.

In the mouse TAA model of ICC, we identified Notch signal activation as a fundamental inducer of hepatocyte conversion into biliary lineage cells at the onset of ICC^8^. When hepatocytes constitutively activated Notch signaling in the liver of TAA-administered mice, the number of hepatocyte-derived biliary lineage cells was significantly increased, resulting in rapid progression to ICC^8^. In contrast, the cell-fate conversion into biliary lineage cells was suppressed in hepatocytes lacking the gene encoding the Notch effector Hes1^8^. Similar to these findings, Fan *et al.*^14^ showed that concomitant activation of Notch and Akt signaling could induce hepatocyte conversion into biliary lineage cells that gave rise to ICC. Moreover, Zender *et al.*^15^ showed that forced activation of Notch signaling in the liver resulted in ICC formation through the upregulation of cyclin E expression, which was identified as a direct transcriptional target of the Notch signaling pathway. Meanwhile, hepatocytes were reported to be capable of conversion into biliary progenitor cells in a mouse model of chronic liver injury induced by 3,5-diethoxycarbonyl-1,4-dihydrocollidine (DDC)^16^,^17^,^18^. In this case, Notch signal activation via the Notch-RBP-Jk-Hes1 signaling axis was critical for the cell-fate conversion of hepatocytes^16^,^17^.

Notch signaling is known to be a key regulator of cholangiocyte differentiation from hepatic progenitor cells during liver development^19^,^20^. Mutations in the JAGGED-1 gene encoding a ligand of the Notch-family receptors or the NOTCH2 gene are responsible for Alagille syndrome, which is characterized by impaired differentiation of cholangiocytes in the developing liver^21^,^22^,^23^,^24^,^25^. Thus, Notch signal activation can be considered a common cue for determination of the fate of cholangiocytes in the liver. During liver organogenesis, Jagged-1 is expressed in endothelial cells and cholangiocytes composing the portal veins (PVs) and bile ducts, respectively^19^, and in periportal mesenchymal cells^26^. Notch signaling might be activated in periportal hepatic progenitor cells and immature biliary cells that express Hes1, leading to their differentiation into cholangiocytes^19^. However, in the injured/diseased adult liver, the mechanisms controlling activation of Notch signaling remain unclear.

In this study, we sought to clarify the machinery for Notch signal activation at the onset of ICC, which is required for induction of hepatocyte conversion into biliary lineage cells, by using the mouse TAA model of ICC. Our present data indicate that Kupffer cells, a resident population of macrophages in the liver, transiently congregate around the central veins (CVs) in the liver and express Jagged-1 soon after the initiation of TAA administration to mice, which is coincident with activation of Notch signaling in pericentral hepatocytes. Moreover, depletion of Kupffer cells prevents the Notch-mediated cell-fate conversion of hepatocytes and leads to death of the TAA-administered mice by inducing hepatocyte apoptosis in their liver. The present findings are useful for understanding how and why hepatocytes change their fate to that of biliary lineage cells at the onset of ICC and may facilitate the development of both prevenient and therapeutic strategies for ICC.

## Results

### Direct conversion of pericentral hepatocytes to biliary lineage cells in the liver of TAA-administered mice

**Figure 1:**
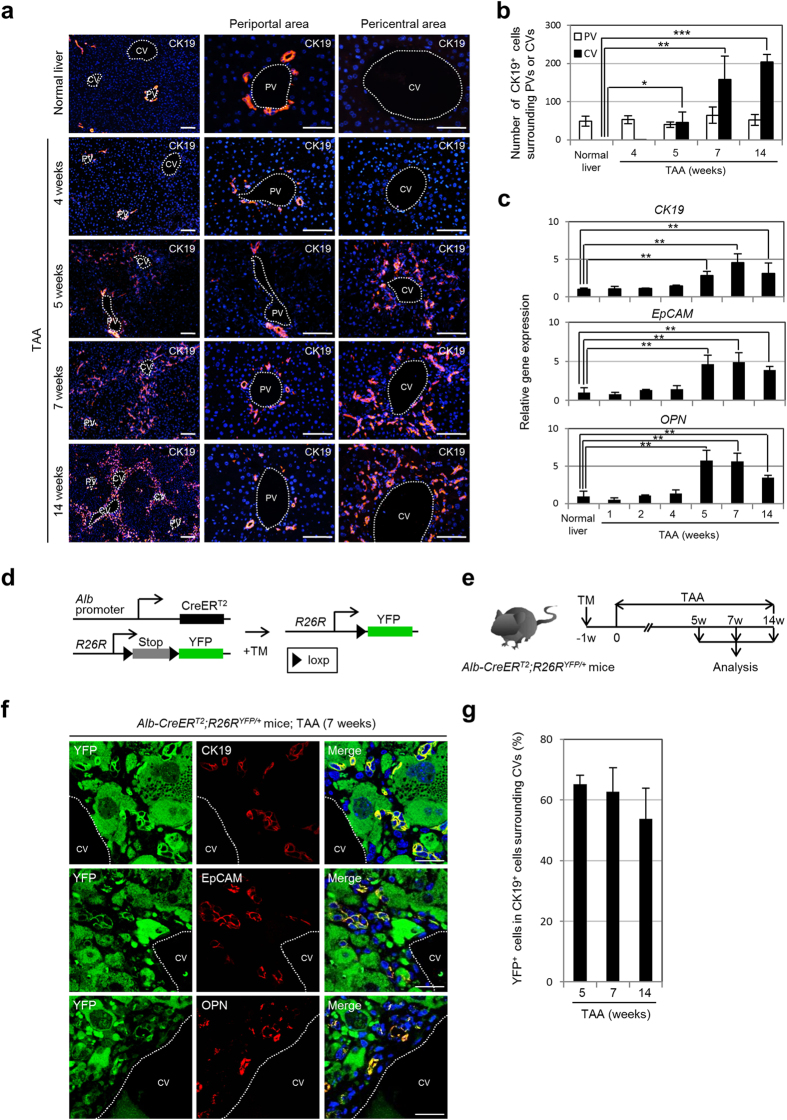
Majority of TAA-induced pericentral biliary lineage cells arise from hepatocytes. (**a**) Immunofluorescence staining of CK19 in the livers of normal and TAA-administered mice. (**b**) Numbers of CK19-positive cells surrounding the PVs or CVs in the livers of normal and TAA-administered mice (25 PVs and 25 CVs per mouse were analyzed in discontinuous liver sections from 3–4 different liver lobes). The data represent means ± SD (*n* = 3). (**c**) RT-qPCR analyses of *CK19*, *EpCAM*, and *OPN* expression were carried out using total RNA derived from the livers of normal and TAA-administered mice. All data were normalized by the value for the internal control gene *Gapdh* and expressed as fold differences from the value in the normal liver. The data represent means ± SD (*n* = 3). (**d**) Experimental procedure to follow the lineage of hepatocytes in the mouse liver. In the presence of TM, CreER^T2^ expressed from the *Alb* genomic locus translocates into the nucleus and removes the loxP-flanked stop cassette from the *R26R* allele, leading to permanent heritable expression of the *YFP* gene. (**e**) Experimental procedure to induce biliary lineage cells around the CVs in the liver. *Alb-CreER*^*T2*^;*R26R*^*YFP*/+^ mice were administered TAA from 1 week after TM injection, and the liver tissues were analyzed after 5, 7, and 14 weeks of TAA administration. w, week(**s**). (**f**) Co-immunofluorescence staining of YFP with CK19, EpCAM, or OPN in the liver of *Alb-CreER*^*T2*^;*R26R*^*YFP*/+^ mice after 7 weeks of TAA administration. (**g**) Percentages of CK19-positive biliary lineage cells co-expressing YFP around the CVs in the liver of *Alb-CreER*^*T2*^;*R26R*^*YFP*/+^ mice after 5, 7, and 14 weeks of TAA administration (10 CVs per mouse were analyzed in discontinuous liver sections from 3–4 different liver lobes). The data represent means ± SD (*n* = 3). DNA was stained with DAPI. Scale bars: 100 μm (**a**) and 25 μm (**f**). *P < 0.05. **P < 0.01. ***P < 0.001.

In the mouse TAA model, cytokeratin (CK) 19-positive biliary lineage cells appear around the CVs in the liver, are distinct from cholangiocytes lining the intrahepatic bile ducts in the portal areas of the hepatic lobule, and contribute to the development of ICC after prolonged exposure to TAA^8^. These newly generated biliary lineage cells were initially observed in the pericentral zone of the hepatic lobule after 5 weeks of TAA administration, and their cell number gradually increased thereafter (Fig. 1a,b). In contrast, the number of cholangiocytes residing around the PVs was maintained at a normal level after TAA administration (Fig. 1a,b). Immunofluorescence analyses revealed that, during TAA treatment, there was only a small change in the number of cholangiocytes expressing Ki-67, a cellular marker for proliferation (Supplemental Fig. 1). The increase in TAA-induced pericentral biliary lineage cells was confirmed by up-regulation of the expression levels of marker genes for biliary lineage cells, such as *CK19*, *epithelial cell adhesion molecule (EpCAM*), and *osteopontin (OPN*), in the liver (Fig. 1c). Interestingly, although the number of Ki-67-positive biliary lineage cells appearing around the CVs reached a peak after 7 weeks of TAA administration, the percentage of these cells was only about 7% (Supplemental Fig. 1). Moreover, the slight increase in Ki-67-positive pericentral biliary lineage cells was transient and returned to a lower level after 14 weeks of TAA administration (Supplemental Fig. 1). Thus, it is suggested that the increase in pericentral biliary lineage cells during TAA treatment arose through different types of cells, rather than by active proliferation of their own cell type.

To identify the source of the TAA-induced biliary lineage cells in the liver, we crossed a mouse line expressing an inducible form of Cre recombinase (*CreER*^*T2*^) from the *albumin (Alb*) genomic locus (*Alb-CreER*^*T2*^ mice)^27^ with a reporter mouse line (*R26R*^*YFP/YFP*^)^28^. In the double-mutant mice, administration of tamoxifen (TM) allowed permanent marking of *Alb*-positive hepatocytes and enabled us to follow the fate of their progeny cells^8^,^17^ (Fig. 1d). In this study, TAA was administered to *Alb-CreER*^*T2*^;*R26R*^*YFP*/+^ mice from 1 week after TM injection to induce biliary lineage cells in the pericentral zone of the hepatic lobule, and the liver tissues were analyzed after 5, 7, and 14 weeks of TAA administration (Fig. 1e). Similar to our previous study^8^, co-immunofluorescence staining of YFP and CK19 revealed that the CK19-positive biliary lineage cells appearing around the CVs in the liver were positive for YFP expression (Fig. 1f). Moreover, these YFP-positive biliary lineage cells were marked by expression of EpCAM and OPN (Fig. 1f). We counted the number of pericentral biliary lineage cells harboring strong fluorescent signals for YFP staining, and found that approximately 60% of CK19-positive cells were specifically marked by YFP expression after 5, 7, and 14 weeks of TAA administration (Fig. 1g). These data indicate that more than half of the TAA-induced pericentral biliary lineage cells were derived from hepatocytes.

Several recent studies have suggested that not only hepatocytes, but also a part of the cholangiocytes composing intrahepatic bile ducts, can respond to the TM-induced Cre activity in *Alb-CreER*^*T2*^ mice^29^,^30^. Thus, in this study, we examined the possibility that the cholangiocytes were also marked by YFP expression in the liver of TM-administered *Alb-CreER*^*T2*^;*R26R*^*YFP*/+^ mice. To identify and exclude false-positive signals caused by auto-fluorescence in the liver and/or non-specific binding of antibodies used for immunofluorescence staining, we compared liver sections obtained from TM-administered age-matched wild-type mice and *Alb-CreER*^*T2*^;*R26R*^*YFP*/+^ mice after staining with an anti-YFP antibody and an Alexa Fluor 488-conjugated secondary antibody. Our data demonstrated that weak fluorescent signals could be detected in hepatocytes, PV endothelial cells, and cholangiocytes in the liver of TM-administered wild-type mice and *Alb-CreER*^*T2*^;*R26R*^*YFP*/+^ mice, indicating that these signals were non-specific background signals (Supplemental Fig. 2a). In contrast, in the liver of TM-administered *Alb-CreER*^*T2*^;*R26R*^*YFP*/+^ mice, we observed strong fluorescent signals in almost all hepatocytes and a part of the cholangiocytes at 1 week after TM administration (Supplemental Fig. 2a). These YFP-positive cholangiocytes sparsely resided in the intrahepatic bile ducts, comprising 6.3 ± 5.6% [mean ± standard deviation (SD)] of the cholangiocytes surrounding each PV in the liver of 5-week-old young adult mice, and further decreasing 2.1 ± 2.4% (mean ± SD) and 2.3 ± 2.8% (mean ± SD) in the liver of 8- and 10-week-old adult mice, respectively (Supplemental Fig. 2a,b). These data suggested that a part of the cholangiocytes in the postnatal mouse liver expressed *Alb*, and that the number of these cells decreased during the progression of liver formation. However, immunofluorescence analyses did not detect any expression of not only intracytoplasmic Alb, but also intranuclear Cre, in the cholangiocytes at 1 day after TM injection in the liver of 5-, 8-, and 10-week-old *Alb-CreER*^*T2*^;*R26R*^*YFP*/+^ mice (Supplemental Fig. 2c,d). Thus, it is suggested that postnatal cholangiocytes can be provided by neighboring *Alb*-positive hepatocytes or hepatic progenitor cells under normal physiological conditions and/or in response to TM-induced hepatic injury, and that the frequency of such provision of cholangiocytes is reduced in accordance with maturation of the liver.

In this study, we normally used adult mice aged 8 weeks or older, in which the number of cholangiocytes marked by unexpected YFP expression was limited. Moreover, during the TAA treatment for 14 weeks, the number of cholangiocytes around the PVs was almost the same as the normal number in pre-treated mice, because of the restricted proliferation of cholangiocytes (Fig. 1a,b and Supplemental Fig. 1). In fact, the percentage of cholangiocytes labeled by unexpected YFP expression in the liver of 8-week-old *Alb-CreER*^*T2*^;*R26R*^*YFP*/+^ mice was not changed after 14 weeks of TAA administration (Supplemental Fig. 2e,f). Thus, the cholangiocytes with strong fluorescent signals for YFP staining in the liver of TM-administered *Alb-CreER*^*T2*^;*R26R*^*YFP*/+^ mice should be maintained as a small population in the intrahepatic bile ducts during TAA treatment, and would not actively contribute to the increase in TAA-induced pericentral biliary lineage cells.

### Transient Notch activation is required for the conversion of pericentral hepatocytes into biliary lineage cells in the TAA-treated mouse liver

Because the toxicity of TAA depends on the activity of cytochrome P450 2B and 2E1 expressed in hepatocytes surrounding the CVs in the liver^31^,^32^,^33^,^34^,^35^, TAA-induced biliary lineage cells may appear as a result of cellular conversion of damaged pericentral hepatocytes. Indeed, the properties of pericentral hepatocytes were rapidly changed after the initiation of TAA treatment. Although pericentral hepatocytes normally expressed glutamine synthetase (GS), but not carbamoyl phosphate synthetase (CPS) 1, the expression levels of GS and CPS1 in pericentral hepatocytes started to decrease and increase, respectively, immediately after the beginning of TAA administration (Supplemental Fig. 3). Moreover, a decrease of the expression levels of the hepatocyte nuclear markers Hnf4α and Tbx3 was also observed in pericentral hepatocytes, soon after the initiation of TAA treatment (Supplemental Fig. 4). These results suggested that the signals required for the deletion of normal hepatocyte features and the conversion of hepatocytes into biliary lineage cells expeditiously stimulated pericentral hepatocytes during the early stage of TAA treatment.

**Figure 2:**
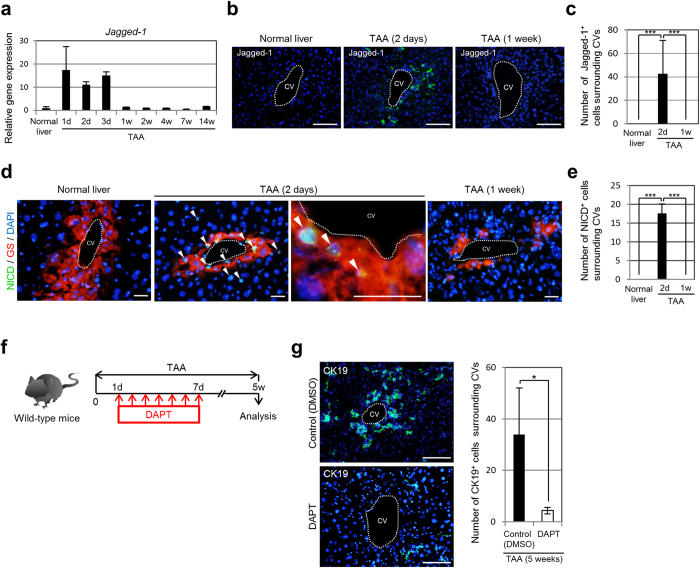
Transient Notch ligand expression is associated with requirement of Notch activation for conversion of pericentral hepatocytes into biliary lineage cells in the liver of TAA-administered mice. (**a**) RT-qPCR analyses of *Jagged-1* expression were carried out using total RNA derived from the livers of normal and TAA-administered mice. All data were normalized by the value for *Gapdh* and expressed as fold differences from the value in the normal liver. The data represent means ± SD (*n* = 3). (**b**) Immunofluorescence staining of Jagged-1 in the livers of normal and TAA-administered mice. (**c**) Numbers of Jagged-1-positive cells surrounding CVs in the livers of normal and TAA-administered mice (10 CVs per mouse were analyzed in discontinuous liver sections from 3–4 different liver lobes). The data represent means ± SD (*n* = 3). (**d**) Co-immunofluorescence staining of the NICD with GS in the livers of normal and TAA-administered mice. NICD-positive hepatocytes (arrowheads) are observed around the CVs after 2 days of TAA administration. (**e**) Numbers of NICD-positive cells surrounding CVs in the livers of normal and TAA-administered mice (10 CVs per mouse were analyzed in discontinuous liver sections from 3–4 different liver lobes). The data represent means ± SD (*n* = 3). (**f**) Experimental procedure to inhibit Notch signal activation during TAA treatment by injecting DAPT into mice. (**g**) Immunofluorescence staining of CK19 in the livers of DMSO-injected (control) and DAPT-injected mice after 5 weeks of TAA administration, and numbers of CK19-positive cells around the CVs in the livers of these mice (20 CVs per mouse were analyzed in discontinuous liver sections from 3–4 different liver lobes). The data represent means ± SD (*n* = 3). DNA was stained with DAPI (blue). Scale bars: 100 μm (**b**,**g**) and 25 μm (**d**). d, day(**s**). w, week(**s**). *P < 0.05. ***P < 0.001.

In our previous study, we found that activation of Notch signaling was essential for induction of the biliary program in pericentral hepatocytes in the liver of TAA-treated mice^8^. Thus, it is possible to speculate that Notch signaling should be activated in hepatocytes surrounding the CVs in the liver soon after the administration of TAA. To address this issue, we investigated the expression of the Notch ligand Jagged-1, which is important for the differentiation of cholangiocytes during liver development by activating Notch signaling in hepatic progenitor cells^19^,^20^. Our data demonstrated that the expression level of *Jagged-1* was rapidly increased at 1, 2, and 3 days after the initiation of TAA administration, but then decreased to a normal level thereafter and was maintained at that level until 14 weeks (Fig. 2a). Immunofluorescence analyses revealed that Jagged-1-positive cells appeared and disappeared in the pericentral zone of the hepatic lobule at 2 days and 1 week after the initiation of TAA treatment, respectively (Fig. 2b). On average, about 40 cells surrounding the CVs in the liver expressed Jagged-1 after 2 days of TAA administration (Fig. 2c). Moreover, the Notch intracellular domain (NICD), which is released from the cell membrane by ligand activation, was found in the nucleus of pericentral hepatocytes at 2 days, but not 1 week, after the initiation of TAA treatment (Fig. 2d,e). These findings suggest that an immediate and transient activation of Notch signaling caused by TAA-induced up-regulation of Jagged-1 expression around the CVs in the liver is important for the induction of conversion of pericentral hepatocytes into biliary lineage cells. Thus, to examine this possibility, we performed daily injections of the γ-secretase inhibitor DAPT into mice to block Notch signal activation from 1 to 7 days after the beginning of TAA administration, and analyzed the liver tissues at 4 weeks after the last injection (Fig. 2f). Immunofluorescence analyses revealed that, in the liver of DAPT-treated mice, the number of CK19-positive biliary lineage cells induced in the pericentral areas of the hepatic lobule was significantly decreased, indicating that a transient activation of Notch signaling within 1 week after the initiation of TAA treatment was essential for the conversion of pericentral hepatocytes into biliary lineage cells (Fig. 2g).

### Kupffer cells transiently express Jagged-1 around the CVs and are required for elevation of *Jagged-1* and *Hes-1* expression in the TAA-treated mouse liver

**Figure 3:**
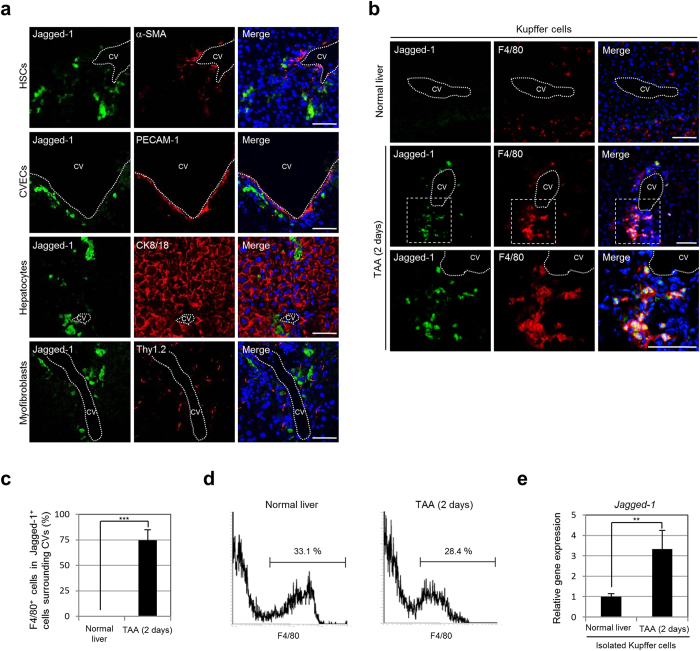
Kupffer cells express Jagged-1 in the pericentral zone of the hepatic lobule after the initiation of TAA treatment. (**a**) Co-immunofluorescence staining of Jagged-1 with alpha-smooth muscle actin (α-SMA) expressed in HSCs, platelet endothelial cell adhesion molecule (PECAM) 1 expressed in CVECs, CK8/18 expressed in hepatocytes, or thymus cell antigen 1 theta (Thy1.2) expressed in myofibroblasts in the liver of mice after 2 days of TAA administration. (**b**) Co-immunofluorescence staining of Jagged-1 with F4/80 expressed in Kupffer cells in the livers of normal and TAA-administered mice. The areas surrounded by the broken lines are enlarged in the bottom panels. (**c**) Percentages of Jagged-1-positive cells co-expressing F4/80 around the CVs in the livers of normal and TAA-administered mice (10 CVs per mouse were analyzed in discontinuous liver sections from 3–4 different liver lobes). The data represent means ± SD (*n* = 3). (**d**) Kupffer cells in NPCs obtained from the livers of normal and TAA-administered mice were fractionated by F4/80 expression and isolated by flow cytometry. The percentages of fractionated cells are shown. Representative data from three independent experiments are shown. (**e**) RT-qPCR analyses of *Jagged-1* expression were carried out using total RNA derived from Kupffer cells isolated from the livers of normal and TAA-administered mice. The data were normalized by the value for *Gapdh* and expressed as fold differences from the value in the normal liver. The data represent means ± SD (*n* = 3). DNA was stained with DAPI (blue). Scale bars: 50 μm. **P < 0.01. ***P < 0.001.

Next, we sought to determine which types of cells expressed Jagged-1 around the CVs in the liver of TAA-administered mice. Immunofluorescence analyses revealed that Jagged-1 was not expressed in hepatic stellate cells (HSCs), CV endothelial cells (CVECs), hepatocytes, and myofibroblasts, but was expressed in Kupffer cells (Fig. 3a,b). Approximately three-fourths of the Jagged-1-positive cells were clearly identified as F4/80-positive Kupffer cells in the pericentral zone of the hepatic lobule after 2 days of TAA administration (Fig. 3c). Moreover, the expression level of *Jagged-1* in Kupffer cells isolated from the liver of mice treated with TAA for 2 days was increased by more than three times compared with that in normal mice (Fig. 3d,e). These findings indicated that Kupffer cells expressed Jagged-1 in response to the TAA-induced liver injury around the CVs in the liver. However, as shown in Fig. 3e, *Jagged-1* expression seems to be detected in Kupffer cells isolated from the normal mouse liver. Thus, it can be speculated that Jagged-1 was also expressed in Kupffer cells residing in the normal mouse liver. However, immunofluorescence analyses revealed that, although Jagged-1 expression was constantly detected in the intrahepatic bile ducts, PVs, and hepatic arteries in the normal mouse liver (Supplemental Fig. 5a,b), no Jagged-1-positive Kupffer cells were found (Supplemental Fig. 5c), suggesting that Jagged-1 expression was post-transcriptionally regulated in Kupffer cells. Alternatively, these findings could suggest that the number of Kupffer cells in the liver increased after TAA administration, and that Jagged-1 was expressed in only a part of the Kupffer cells residing around the CVs in the TAA-treated liver. Indeed, at 2 days after TAA administration, we observed an increase in the number of Kupffer cells in the liver, which subsequently returned to a normal level within 2 weeks of TAA administration (Supplemental Fig. 6a). Moreover, Kupffer cells were congregated around the CVs in the liver of mice treated with TAA for 2 days, but started to return to a normal distribution pattern between 1 and 2 weeks after the initiation of TAA administration (Supplemental Fig. 6a,b).

**Figure 4:**
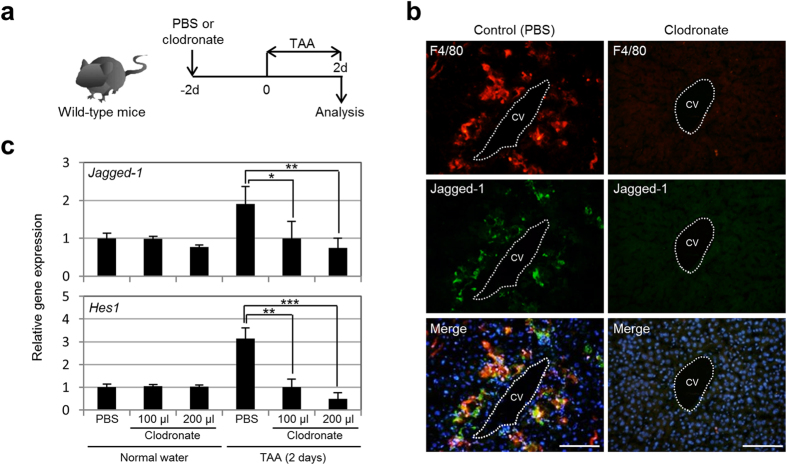
Notch signal activation in the TAA-treated mouse liver depends on the presence of Kupffer cells. (**a**) Experimental procedure to induce depletion of Kupffer cells from the TAA-treated mouse liver. We injected PBS (control) or clodronate into mice at 2 days before the beginning of TAA administration, and analyzed the liver tissues after 2 days of TAA administration. d, days. (**b**) Co-immunofluorescence staining of F4/80 with Jagged-1 in the livers of PBS-injected and clodronate-injected mice after 2 days of TAA administration. DNA was stained with DAPI (blue). Scale bars: 100 μm. (**c**) RT-qPCR analyses of *Jagged-1* and *Hes1* expression were carried out using total RNA derived from the livers of PBS-injected and clodronate-injected normal and TAA-administered mice. All data were normalized by the value for *Gapdh* and expressed as fold differences from the value in the normal liver. The data represent means ± SD (*n* = 3). *P < 0.05. **P < 0.01. ***P < 0.001.

Given the above findings, we further investigated whether Notch signal activation in the TAA-treated mouse liver was dependent on the presence of Kupffer cells. To address this issue, we depleted Kupffer cells in the liver by injecting clodronate to induce apoptosis of Kupffer cells in the mice at 2 days before the beginning of TAA administration (Fig. 4a). After 2 days of TAA administration, the numbers of Kupffer cells and Jagged-1-positive cells around the CVs in the liver of the clodronate-injected mice were significantly reduced, and the expression levels of *Jagged-1* and *Hes1* in the liver of these mice became normal levels (Fig. 4b,c). Thus, our present data demonstrate that Kupffer cells are rapidly increased in response to TAA-induced liver injury, move to congregate in the pericentral zone of the hepatic lobule within 2 days, and remain there for 1 week to potentially induce Notch signal activation in neighboring hepatocytes.

### Kupffer cell-associated Notch ligand expression is characteristic for the TAA-induced conversion of pericentral hepatocytes into biliary lineage cells

Cell-fate conversion of hepatocytes into biliary lineage cells has also been considered as a possible phenomenon in the response to other types of liver injury, including the ductular reaction induced in the periportal zone of the hepatic lobule by administration of DDC to adult mice^16^,^17^,^18^. Similar to the case for TAA, DDC induced hepatocyte conversion into biliary lineage cells by activating Notch signaling in the liver^16^,^17^. Thus, to examine whether Notch signal activation by Jagged-1-expressing Kupffer cells is a common mechanism in the hepatotoxin-induced conversion of hepatocytes into biliary lineage cells, we conducted immunofluorescence analyses to uncover the machinery for Notch signal activation in the ductular reaction caused by DDC administration.

During 3 weeks of DDC treatment, the number of biliary lineage cells forming the biliary ductules gradually increased around the PVs, but not the CVs, in the liver, and the expression levels of *Jagged-1* and *Hes1* in the liver increased in accordance with the period of DDC administration (Supplemental Fig. 7). Moreover, the NICD was found in the periportal, but not pericentral, hepatocytes after 1 week of DDC treatment (Supplemental Fig. 8). During induction of the ductular reaction by DDC for 3 weeks, Kupffer cells gradually congregated around the PVs in the liver, suggesting that Kupffer cells moved to accumulate in the area damaged by DDC administration, similar to the pericentral region that was specifically injured by TAA (Supplemental Fig. 9). In the case of DDC-induced liver damage, Jagged-1 expression was clearly detected in the periportal, but not pericentral, zone of the hepatic lobule (Supplemental Fig. 10a). However, Jagged-1 was commonly expressed in cholangiocytes lining the intrahepatic bile ducts, endothelial cells composing the PVs and hepatic arteries, and smooth muscle cells surrounding endothelial cells, but never in Kupffer cells, which resided in both the normal and DDC-treated mouse liver (Supplemental Figs 5, 10 and 11). Thus, our findings indicate that Kupffer cell-associated Notch ligand expression is characteristic for the TAA-induced conversion of hepatocytes into biliary lineage cells around the CVs in the liver.

### Kupffer cells block the apoptosis of pericentral hepatocytes and induce their conversion into biliary lineage cells in the liver of TAA-administered mice

**Figure 5:**
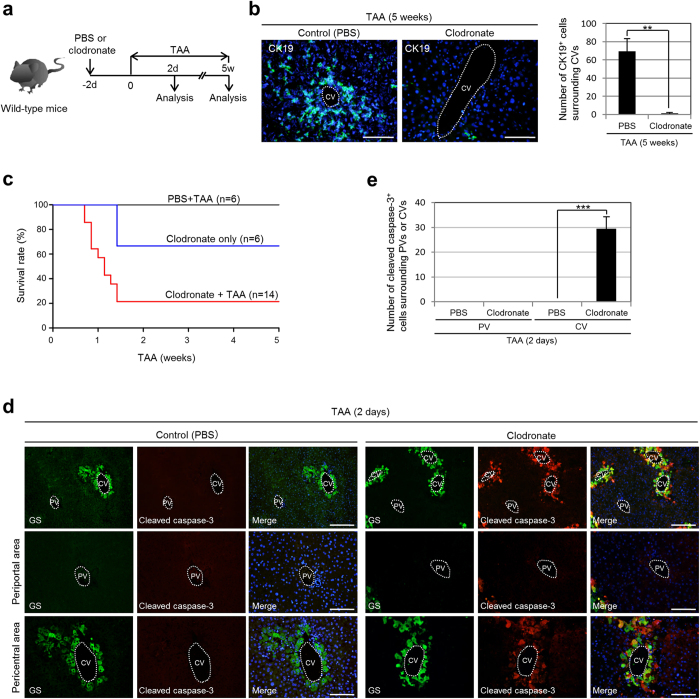
Kupffer cells block the apoptosis of pericentral hepatocytes and induce their conversion into biliary lineage cells in the TAA-treated mouse liver. (**a**) Experimental procedure to examine the effect of Kupffer cell depletion in the liver during TAA treatment. We injected PBS (control) or clodronate into mice at 2 days before the beginning of TAA administration, and analyzed the liver tissues after 2 days and 5 weeks of TAA administration. d, days. w, weeks. (**b**) Immunofluorescence staining of CK19 in the livers of PBS-injected and clodronate-injected mice after 5 weeks of TAA administration, and numbers of CK19-positive cells around the CVs in the livers of these mice (25 CVs per mouse were analyzed in discontinuous liver sections from 3–4 different liver lobes). The data represent means ± SD (*n* = 3). (**c**) Kaplan–Meier survival curves of mice injected with PBS or clodronate, with or without subsequent TAA administration. (**d**) Co-immunofluorescence staining of GS with cleaved caspase-3 in the livers of PBS-injected and clodronate-injected mice after 2 days of TAA administration. (**e**) Numbers of cleaved caspase-3-positive cells residing around the PVs and CVs in the livers of PBS-injected and clodronate-injected mice after 2 days of TAA administration (10 PVs and 10 CVs per mouse were analyzed in discontinuous liver sections from 3–4 different liver lobes). The data represent means ± SD (*n* = 3). DNA was stained with DAPI (blue). Scale bars: 100 μm. **P < 0.01. ***P < 0.001.

Finally, we examined whether Kupffer cells are required to induce the conversion of hepatocytes into biliary lineage cells in the liver of TAA-administered mice. To this end, we injected clodronate into the mice at 2 days before the beginning of TAA administration and analyzed the liver tissues after 5 weeks (Fig. 5a). Following injection of clodronate into the mice, Kupffer cells were efficiently depleted from the liver, but the number of these cells gradually increased from 7 days after the injection and completely recovered by 10 days after the injection (Supplemental Fig. 12). Thus, in this experiment, the function of Kupffer cells could be suppressed for 1 week after the clodronate injection. Immunofluorescence analyses revealed that, in the liver of clodronate-injected mice, the number of CK19-positive biliary lineage cells induced in the pericentral areas of the hepatic lobule was significantly decreased after 5 weeks of TAA administration (Fig. 5b). These findings indicate that, to induce the conversion of hepatocytes into biliary lineage cells, Kupffer cells should be present in the liver until 5 days after the initiation of TAA treatment. This result is consistent with our data showing that a transient activation of Notch signaling within 1 week after the beginning of TAA administration was required for the subsequent conversion of hepatocytes into biliary lineage cells in the pericentral zone of the hepatic lobule (Fig. 2f,g). These data suggest that pericentral hepatocytes can be converted into biliary lineage cells as a result of a transient activation of Notch signaling induced by Kupffer cells expressing Jagged-1 for only several days after the initiation of TAA administration.

Interestingly, the mice injected with clodronate immediately started to die from 5 days after the beginning of TAA administration. Survival curves revealed that only 20% of clodronate-injected mice survived beyond 5 weeks of TAA administration (Fig. 5c). While clodronate itself was toxic toward the injected mice, TAA administration after the injection of clodronate significantly increased the mortality of the mice. By analyzing the liver tissues of the clodronate-injected mice at 2 days after initiation of TAA treatment, we found a marked increase in apoptotic hepatocytes in the pericentral zone of the hepatic lobule (Fig. 5d,e). Thus, in the TAA-treated mouse liver, Kupffer cells act as a suppressor for the apoptosis of pericentral hepatocytes and induce their conversion into biliary lineage cells. Moreover, our data suggest that, in the absence of Kupffer cells, pericentral hepatocytes cannot survive under the toxicity of TAA, which may arise through the lack of Notch signal activation and lead to the death of the mice.

## Discussion

**Figure 6:**
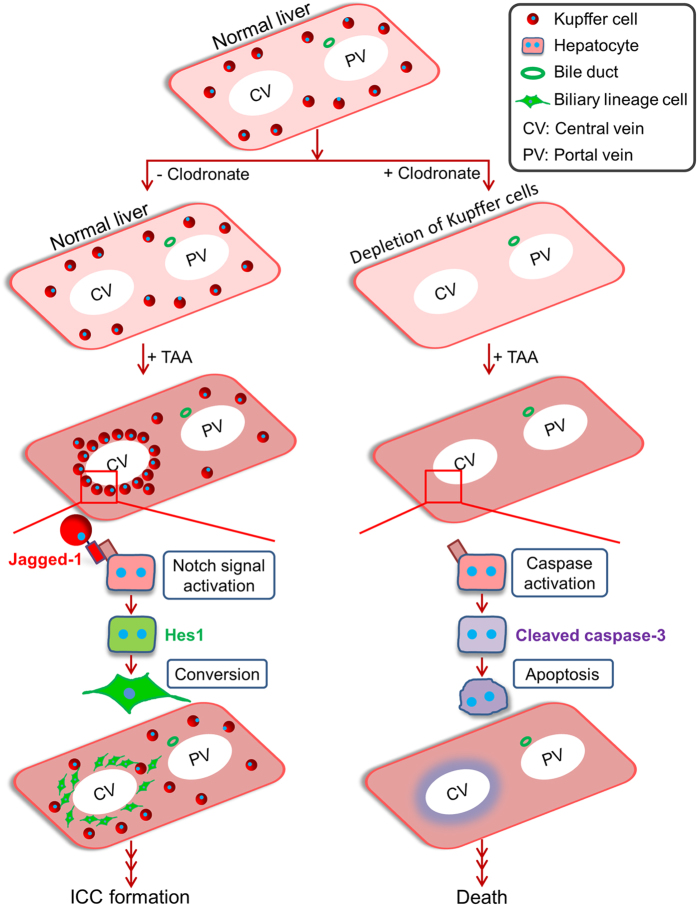
Scheme for the roles of Kupffer cells in the TAA-treated mouse liver. In response to TAA-induced liver injury, Kupffer cells transiently congregate around the CVs in the liver and express Jagged-1, which induces activation of Notch signaling in pericentral hepatocytes and leads to their conversion into biliary lineage cells. A lack of Kupffer cells in the liver prevents the Notch-mediated cell-fate conversion of hepatocytes and increases mortality in mice by inducing the apoptosis of pericentral hepatocytes.

Elucidation of the pathogenic mechanism of ICC is important for developing methods for prophylaxis and treatment of this malignant tumor in the liver. However, our incomplete understanding of the pathogenesis of ICC prevents improvements in the therapeutic outcome of patients with ICC. In this study, we found that, in the mouse TAA model of ICC, Kupffer cells are immediately, but transiently, assembled to the pericentral zone of the hepatic lobule and express Jagged-1 in response to the TAA-induced hepatic injury, which leads to Notch signal activation in pericentral hepatocytes and their subsequent conversion into biliary lineage cells that become a source of ICC (Fig. 6). Moreover, Kupffer cells are required to block the TAA-induced apoptosis of pericentral hepatocytes, which results in a significant decrease in mortality of the mice (Fig. 6). Thus, the roles of Kupffer cells in this mouse model of ICC can be considered as a double-edged sword, with the cells being essential for not only the survival of TAA-administered mice, but also the generation of a cellular origin of ICC by inducing conversion of hepatocytes into biliary lineage cells.

The roles of Kupffer cells in liver injury have been analyzed in previous studies. It has been shown that Kupffer cells express Wnt3a to activate canonical Wnt signaling and inhibit Notch signaling in hepatic progenitor cells for specification to hepatocytes in the liver of mice administered a choline-deficient, ethionine-supplemented diet, leading to periportal lesions in the liver^36^. The significance of Kupffer cells has also been reported in the rat TAA model of ICC, in which Kupffer cells express Wnt7b to activate canonical Wnt signaling at a later stage of TAA treatment and are involved in the progression of ICC^37^. Meanwhile, the present data demonstrate that Kupffer cells express Jagged-1 coincident with Notch activation in pericentral hepatocytes in the liver of TAA-administered mice, but never express Jagged-1 under the periportal damage caused by DDC administration. Thus, it is suggested that Kupffer cells can differentially respond to hepatic injury in accordance with the region of injury in the hepatic lobule and the characteristic of the hepatotoxin involved. During the process of ICC formation, Kupffer cells may have various roles, including Notch signal activation in hepatocytes at the onset of ICC, as shown in this study, and Wnt signal activation in biliary lineage cells composing the progressive ICC^37^. For future clinical applications, the properties of Kupffer cells in liver injury and tumors need to be clarified, and then regulated in a spatio-temporal manner.

Notch signal activation can be considered a common stimulus for determination of the biliary fate in both the developing liver and the injured/diseased adult liver. However, the downstream targets of Notch signaling that allow the biliary fate decision remain largely unknown. In mouse and human ICC, the expression level of *cyclin E* is directly upregulated by activation of the Notch signaling pathway, and the suppression of this expression inhibits tumor formation^15^. Thus, cyclin E can be identified as a downstream effector of Notch signaling for ICC formation. However, because Notch signal activation is suggested to have an anti-proliferative effect on hepatocytes, the Notch-induced upregulation of cyclin E expression may be involved in the development and progression of ICC, but not in the process of hepatocyte conversion into biliary lineage cells^15^,^38^. Thus, Notch signaling may contribute to various stages of ICC formation through the use of different downstream targets depending on the time and place during tumorigenesis. Since the cell-fate conversion into biliary lineage cells is suppressed in Hes1-deleted mouse hepatocytes, the transcriptional targets of Hes1 may contain the genes essential for induction of the biliary fate^8^,^17^.

In the mouse TAA model of ICC, our lineage-tracing analyses revealed that pericentral hepatocytes could change their fate to that of biliary lineage cells and become a cellular origin of ICC. In our previous study, we administered TM into *Alb-CreER*^*T2*^;*R26R*^*YFP*/+^ mice by intraperitoneal injection to label the hepatocytes with YFP expression. Although this protocol enabled us to specifically mark the hepatocytes with YFP expression, the mortality rate of the TM-injected mice was very high. Thus, in the present study, we changed the method of TM administration to subcutaneous injection, after which almost all mice survived without losing the efficient marking of hepatocytes with YFP expression. However, in the liver of *Alb-CreER*^*T2*^;*R26R*^*YFP*/+^ mice subcutaneously injected with TM, we observed that the intrahepatic bile ducts contained a small number of cholangiocytes marked with unexpected expression of YFP. These results suggest that data obtained from TM-mediated lineage-tracing experiments are dependent on the protocol of TM injection, which is probably based on the degree of difference in the TM-induced liver injury. Thus, in the lineage-tracing experiments using TM, detailed analyses are strongly required for correct understanding, as we showed in this study.

The expression of marker genes used for lineage-tracing analyses needs to be carefully examined. Recent studies demonstrated that a part of the periportal hepatocytes expressed Sox9, which is generally known as a marker of cholangiocytes but not hepatocytes in the liver, and these Sox9-positive hepatocytes were designated hybrid hepatocytes^29^. The finding of hybrid hepatocytes suggests that other biliary marker genes may also be expressed in these cells. Indeed, in addition to Sox9, periportal hepatocytes expressed another cholangiocyte marker, Hnf1β, in the normal mouse liver, and the expression levels of these proteins were slightly increased at 1 day after TM injection (Supplemental Fig. 13). Thus, previous data obtained from lineage-tracing analyses of cholangiocytes based on the expression levels of Sox9 and Hnf1β may require reconsideration^30^,^39^. Meanwhile, unexpected reporter gene expression also needs to be considered for precise interpretation of lineage-tracing experiments. As shown in this study, YFP expression can be found not only in hepatocytes, but also in a small number of cholangiocytes, in the liver of TM-administered *Alb-CreER*^*T2*^;*R26R*^*YFP*/+^ mice. Our data suggest that these YFP-positive cholangiocytes were derived from *Alb*-positive hepatocytes or hepatic progenitor cells residing around the portal areas of the hepatic lobule, which arose in a spontaneous manner and/or in response to the TM-induced hepatic injury.

Other recent reports have suggested a contribution of cholangiocytes to the increase in TAA-induced biliary lineage cells in the pericentral zone of the hepatic lobule, as a result of migration of a part of the cholangiocytes from the intrahepatic bile ducts^40^,^41^. In this study, we observed a small number of protrusions formed by a population of biliary lineage cells in the liver of TM-injected *Alb-CreER*^*T2*^;*R26R*^*YFP*/+^ mice after 14 weeks of TAA administration, while these cells were totally negative for YFP expression (Supplemental Fig. 14). Thus, there is a possibility that, to a lesser extent, the cholangiocytes lining the intrahepatic bile ducts can contribute to the increase in TAA-induced biliary lineage cells by migrating from the portal areas to the central areas of the hepatic lobule. In the present study, about 40% of TAA-induced pericentral biliary lineage cells were identified as cells negative for YFP expression in the liver of *Alb-CreER*^*T2*^;*R26R*^*YFP*/+^ mice (Fig. 1g). Thus, it is suggested that cells other than hepatocytes, including cholangiocytes, can partially contribute to the increase in pericentral biliary lineage cells during TAA treatment, or that these YFP-negative biliary lineage cells may appear through a limitation of TM-inducible Cre activity and/or accidental gene silencing involved in YFP expression, in the liver of TM-administered *Alb-CreER*^*T2*^;*R26R*^*YFP*/+^ mice.

Hepatocytes are generally considered to be one of the terminally differentiated cells in adult organisms, with metabolic functions and no lineage plasticity. However, our genetic lineage-tracing experiments revealed that hepatocytes can be converted into biliary lineage cells in response to activation of Notch signaling that is specifically induced in the injured/diseased liver. Thus, it is suggested that hepatocytes can act as an alternative to hepatic stem cells capable of replicating their own cell population and giving rise to biliary lineage cells. Meanwhile, recent studies have provided another possibility that both periportal and pericentral hepatocytes are distinguished from other hepatocytes, originally possess the properties of hepatic stem/progenitor cells, and may enable the production of biliary lineage cells by direct differentiation through Notch signal activation^29^,^42^. In any case, precise analyses and deep understanding of the events that occur in the region where biliary lineage cells are generated from hepatocytes in the injured/diseased liver will facilitate the development of methods for the diagnosis, prevention, and treatment of ICC.

## Methods

### Mice

C57BL/6 mice (Clea), *Alb-CreER*^*T2*^ mice (a gift from Pierre Chambon and Daniel Metzger, Institute of Genetics and Molecular and Cellular Biology, Illkirch, France), and *R26R*^*YFP/YFP*^ mice (a gift from Frank Costantini, Columbia University, New York, NY, USA) were used in this study. All animal experiments were approved by the Kyushu University Animal Experiment Committee, and the care and use of the animals were performed in accordance with institutional guidelines.

### Chemical administration

For induction of Cre activity, mice (8–10 weeks of age) were given a single subcutaneous injection of TM (7.5 mg/mouse; Sigma-Aldrich) dissolved in olive oil (Nacalai Tesque) at a concentration of 50 mg/ml. To induce liver injury, the mice were provided with drinking water containing TAA (300 mg/l; Wako)^8^ or a diet containing 0.1% DDC (Sigma-Aldrich)^43^. For Notch signal inhibition, the mice were given intraperitoneal injections of DAPT (50 mg/kg; Wako) dissolved in dimethyl sulfoxide (Nacalai Tesque)/olive oil (1:9) mixed solvent or vehicle alone daily for 1 week. For Kupffer cell ablation, the mice received a single intravenous injection of 100 or 200 μl of clodronate liposomes (Hygieia Bioscience) or control phosphate-buffered saline (PBS).

### Antibody generation

The CK19 antigen was synthesized based on its specific amino acid sequence HYNNLPTPKAI (Genenet Co.), as described previously^8^. The peptide was conjugated to keyhole limpet hemocyanin with maleimide (Thermo Scientific), a carrier protein. Rat anti-mouse CK19 monoclonal antibodies were generated based on the lymph node method^44^.

### Immunostaining

For paraffin-embedded tissue sections, liver tissues were fixed in 20% formalin, dehydrated in ethanol and xylene, embedded in paraffin wax, and sectioned. After deparaffinization and rehydration of the sections, antigen retrieval was performed by microwaving in 0.01 M citrate buffer (pH 6.0). For frozen tissue sections, liver tissues were directly embedded in Tissue-Tek OCT optimal cutting temperature compound (Sakura Finetek) and sectioned. The frozen tissue sections were initially fixed with 4% paraformaldehyde for 5 minutes and then fixed with methanol for 5 minutes at room temperature. After washing in PBS containing 0.1% Tween-20 and blocking, the sections were incubated with the primary antibodies shown in Supplemental Table 1. After washing, the sections were incubated with Alexa 488-, Alexa 555-, and/or Alexa 647-conjugated secondary antibodies (1:1000; Life Technologies) with DAPI. Stained tissues were viewed using an Olympus IX71 microscope and an Olympus FluoView FV10i confocal laser-scanning microscope.

### Kupffer cell isolation

Single cell suspensions of hepatic non-parenchymal cells (NPCs) were prepared from the liver of mice with or without TAA treatment for 2 days by excluding hepatocytes from the total liver cells isolated by two-step collagenase digestion^45^. The Kupffer cells were then labeled with an Alexa Fluor 647-conjugated anti-F4/80 monoclonal antibody (AbD Serotec) and isolated using a FACS Jazz cell sorter (BD Biosciences).

### Reverse transcription-quantitative polymerase chain reaction (RT-qPCR) analysis

Total RNA was prepared from the liver tissues and Kupffer cells using NucleoSpin RNA II (Takara Bio) according to the manufacturer’s instructions, and cDNAs were synthesized from the total RNA as described^46^. RT-qPCR was conducted as described previously^47^. Details regarding the qPCR primers and probes were provided in a previous report^47^, except for the qPCR primer/probes for *EpCAM* (Mm00493214_m1), *OPN* (Mm00436767_m1), *Jagged-1* (Mm00496902_m1), and *Hes1* (Mm01342805_m1), cited with TaqMan Gene Expression Assay IDs (Applied Biosystems) in parentheses following the names of the genes. To examine the expression of glyceraldehyde-3-phosphate dehydrogenase (*Gapdh*) as a normalization control, we used TaqMan Rodent GAPDH Control Reagents (Applied Biosystems).

### Statistics

Statistical significance was analyzed using an unpaired Student’s *t*-test. A difference at *P* < 0.05 was considered statistically significant.

## Additional Information

**How to cite this article**: Terada, M. *et al.* Kupffer cells induce Notch-mediated hepatocyte conversion in a common mouse model of intrahepatic cholangiocarcinoma. *Sci. Rep.* **6**, 34691; doi: 10.1038/srep34691 (2016).

## Supplementary Material

Supplementary Information
