# Supplementary material for: Kupffer cells induce Notch-mediated hepatocyte conversion in a common mouse model of intrahepatic cholangiocarcinoma
Source: Sci Rep. 2016 Oct 4;6:34691. doi: 10.1038/srep34691 (PMC5048166; doi:10.1038/srep34691)
Supplement: Supplementary Information [file srep34691-s1.pdf]

Supplementary Information

Kupffer cells induce Notch-mediated hepatocyte conversion in a common mouse model of intrahepatic cholangiocarcinoma

Maiko Terada[1], Kenichi Horisawa[1], Shizuka Miura[1], Yasuo Takashima[1], Yasuyuki Ohkawa[2, 3], Sayaka Sekiya[1], Kanae Matsuda-Ito[1], Atsushi Suzuki[1, 3,*]

[1]Division of Organogenesis and Regeneration, Medical Institute of Bioregulation, Kyushu University, 3-1-1 Maidashi, Higashi-ku, Fukuoka 812-8582, Japan

[2]Division of Transcriptomics, Medical Institute of Bioregulation, Kyushu University, 3-1-1 Maidashi, Higashi-ku, Fukuoka 812-8582, Japan

[3]Core Research for Evolutional Science and Technology, The Japan Agency for Medical Research and Development, 1-7-1 Otemachi, Chiyoda-ku, Tokyo 100-0004, Japan

*Corresponding author: Atsushi Suzuki, Division of Organogenesis and Regeneration, Medical Institute of Bioregulation, Kyushu University, 3-1-1 Maidashi, Higashi-ku,

Fukuoka 812-8582, Japan.

Tel/Fax: +81-92-642-6784; E-mail: suzukicks@bioreg.kyushu-u.ac.jp

Supplemental figure legends

Supplemental Figure 1. Number of Ki-67-positive pericentral biliary lineage cells is transiently, but not significantly, increased during TAA treatment. Co-immunofluorescence staining of Ki-67 with CK19 was conducted in the livers of normal and TAA-administered mice, and the percentages of CK19-positive cells co-expressing Ki-67 in the periportal and pericentral areas of the hepatic lobule were calculated (15 PVs and 15 CVs per mouse were analyzed in discontinuous liver sections from 3–4 different liver lobes). The data represent means ± SD (*n* = 3). The areas surrounded by the broken line were enlarged. CK19-positive cells co-expressing Ki-67 are indicated by arrowheads. DNA was stained with DAPI (blue). Scale bars: 100 µm.

Supplemental Figure 2. Analysis of YFP-positive cholangiocytes found in the liver of TM-administered *Alb-CreER*^{T2};*R26R*$^{YFP/+}$ mice. (a) Co-immunofluorescence staining of YFP with CK19 in the livers of 10-week-old wild-type mice and 5-, 8-, and 10-week-old *Alb-CreER*^{T2};*R26R*$^{YFP/+}$ mice at 1 week after TM injection. CK19-positive

cholangiocytes co-expressing YFP are indicated by arrowheads. (b) Percentages of CK19-positive cholangiocytes co-expressing YFP in the livers of 5-, 8-, and 10-week-old *Alb-CreER*^{T2};*R26R*$^{YFP/+}$ mice at 1 week after TM injection. The data represent means (red bars) ± SD (n = 2). (c) Co-immunofluorescence staining of YFP with Alb (upper panels) or Cre (lower panels) in the liver of 5-week-old *Alb-CreER*^{T2};*R26R*$^{YFP/+}$ mice at 1 day after TM injection. YFP-positive cholangiocytes are indicated by arrowheads. (d) Percentages of CK19-positive cholangiocytes co-expressing Alb (upper graph) or Cre (lower graph) in the livers of 5-, 8-, and 10-week-old *Alb-CreER*^{T2};*R26R*$^{YFP/+}$ mice at 1 day after TM injection. The data represent means (red bars) ± SD (n = 3). (e) Co-immunofluorescence staining of YFP with CK19 in the liver of TM-injected 8-week-old *Alb-CreER*^{T2};*R26R*$^{YFP/+}$ mice after 14 weeks of TAA administration. The area surrounded by the broken line was enlarged. A CK19-positive cholangiocyte co-expressing YFP is indicated by an arrowhead. (f) Percentages of CK19-positive cholangiocytes co-expressing YFP in the liver of TM-injected 8-week-old *Alb-CreER*^{T2};*R26R*$^{YFP/+}$ mice after 5, 7, and 14 weeks of TAA administration. The data represent means (red bars) ± SD (n = 3). DNA was stained with

DAPI (blue). Scale bars: 20 µm (a and c) and 50 µm (e). Fifteen PVs per mouse were analyzed in discontinuous liver sections from 3–4 different liver lobes (b, d, and f). d, day. w, week(s).

Supplemental Figure 3. Properties of pericentral hepatocytes immediately change after the beginning of TAA administration. Co-immunofluorescence staining of GS with CPS1 was conducted in the livers of normal and TAA-administered mice. DNA was stained with DAPI (blue). Scale bars: 100 µm.

Supplemental Figure 4. The expression levels of Hnf4α and Tbx3 in pericentral hepatocytes start to decrease, immediately after the beginning of TAA administration. (a) Co-immunofluorescence staining of Hnf4α with CK8/18 was conducted in the livers of normal and TAA-administered mice. (b) Co-immunofluorescence staining of Tbx3 with N-cadherin was conducted in the livers of normal and TAA-administered mice. DNA was stained with DAPI (blue). Scale bars: 100 µm.

Supplemental Figure 5. Jagged-1 is expressed in cholangiocytes lining the intrahepatic bile ducts, endothelial cells composing the PVs and hepatic arteries, and smooth muscle cells surrounding endothelial cells, but not in Kupffer cells, in the normal mouse liver. (a) Co-immunofluorescence staining of CK19 with PECAM-1 in the normal mouse liver. The area surrounded by the broken line was enlarged. Note that cholangiocytes and endothelial cells can be individually stained. (b) Co-immunofluorescence staining of Jagged-1 with CK19 (upper panels) or PECAM-1 (lower panels) in the normal mouse liver. The areas surrounded by the broken line were enlarged. Note that Jagged-1 is expressed in not only cholangiocytes and endothelial cells, but also smooth muscle cells surrounding endothelial cells. (c) Co-immunofluorescence staining of Jagged-1 with F4/80 in the normal mouse liver. DNA was stained with DAPI (blue). Scale bars: 20 µm (a and b) and 100 µm (c).

Supplemental Figure 6. Kupffer cells transiently congregate around the CVs in the liver of TAA-administered mice. (a) Immunofluorescence staining of F4/80 in the livers of normal and TAA-administered mice. DNA was stained with DAPI (blue). Scale bars:

100 µm. (b) Numbers of F4/80-positive cells surrounding the PVs or CVs in the livers of normal and TAA-administered mice (10 PVs and 10 CVs per mouse were analyzed in discontinuous liver sections from 3–4 different liver lobes). The data represent means ± SD (n = 3). d, days. w, week(s). ***P < 0.001.

Supplemental Figure 7. DDC treatment induces biliary ductule formation around the PVs and increases the expression levels of *Jagged-1* and *Hes1* in the liver. (a) Immunofluorescence staining of CK19 in the livers of normal and DDC-administered mice. DNA was stained with DAPI (blue). Scale bars: 100 µm. (b) RT-qPCR analyses of *Jagged-1* and *Hes1* expression were carried out using total RNA derived from the livers of normal and DDC-administered mice. All data were normalized by the value for *Gapdh* and expressed as fold differences from the value in the normal mouse liver (n = 3). **P < 0.01. ***P < 0.001.

Supplemental Figure 8. DDC treatment activates Notch signaling in periportal hepatocytes in the liver. (a) Trichrome immunofluorescence staining of the NICD with

CK8/18 and CK19 was conducted in the livers of mice fed with a normal diet or DDC-containing diet for 1 week. NICD-positive hepatocytes (arrowheads) expressing CK8/18, but not CK19, are observed around the PVs after 1 week of DDC administration. (b) Co-immunofluorescence staining of NICD with Hnf4α was conducted in the liver of DDC-administered mice. Hnf4α-positive hepatocytes co-expressing NICD are indicated by arrowheads. DNA was stained with DAPI (blue). Scale bars: 50 μm (a and b).

Supplemental Figure 9. Kupffer cells congregate around the PVs in the liver during DDC treatment. Immunofluorescence staining of F4/80 was conducted in the livers of normal and DDC-administered mice. DNA was stained with DAPI (blue). Scale bars: 100 μm.

Supplemental Figure 10. Expression pattern of Jagged-1 is not changed between the normal and DDC-treated mouse livers. (a) Immunofluorescence staining of Jagged-1 was conducted in the livers of normal and DDC-administered mice. (b)

Co-immunofluorescence staining of Jagged-1 with CK19 in the liver of DDC-administered mice. (c) Co-immunofluorescence staining of Jagged-1 with PECAM-1 in the liver of DDC-administered mice. Note that Jagged-1 is expressed in both endothelial cells and smooth muscle cells surrounding endothelial cells. DNA was stained with DAPI (blue). Scale bars: 100 µm (a) and 20 µm (b and c).

Supplemental Figure 11. Kupffer cells do not express Jagged-1 in both the normal and DDC-treated mouse livers. Co-immunofluorescence staining of Jagged-1 with F4/80 was conducted in the livers of normal and DDC-administered mice. DNA was stained with DAPI (blue). Scale bars: 20 µm.

Supplemental Figure 12. Kupffer cells are absent in the liver for 1 week after a single injection of clodronate into mice. (a) Experimental procedure to examine the effect of clodronate on Kupffer cell depletion. d, day(s). (b) Immunofluorescence staining of F4/80 in the livers of normal and clodronate-injected mice. DNA was stained with DAPI (blue). Scale bars: 100 µm.

Supplemental Figure 13. Cholangiocyte marker expression is detected in periportal hepatocytes in both the normal and TM-injected mouse livers. (a and b) Co-immunofluorescence staining of Hnf4α with Sox9 (a) or Hnf1β (b) in the livers of normal and TM-injected mice. Hnf4α-positive hepatocytes co-expressing Sox9 or Hnf1β are indicated by arrowheads. DNA was stained with DAPI (blue). Scale bars: 20 μm. d, day.

Supplemental Figure 14. Small number of protrusions formed by a population of biliary lineage cells in the liver of TAA-administered mice are negative for YFP expression. Co-immunofluorescence staining of YFP with EpCAM was conducted in the liver of *Alb-CreER*^{T2};*R26R*$^{YFP/+}$ mice after 14 weeks of TAA administration. DNA was stained with DAPI (blue). Scale bars: 50 μm.

Supplemental Table 1. List of the primary antibodies used for immunofluorescence staining.

Terada et al., Supplementary Figure 1

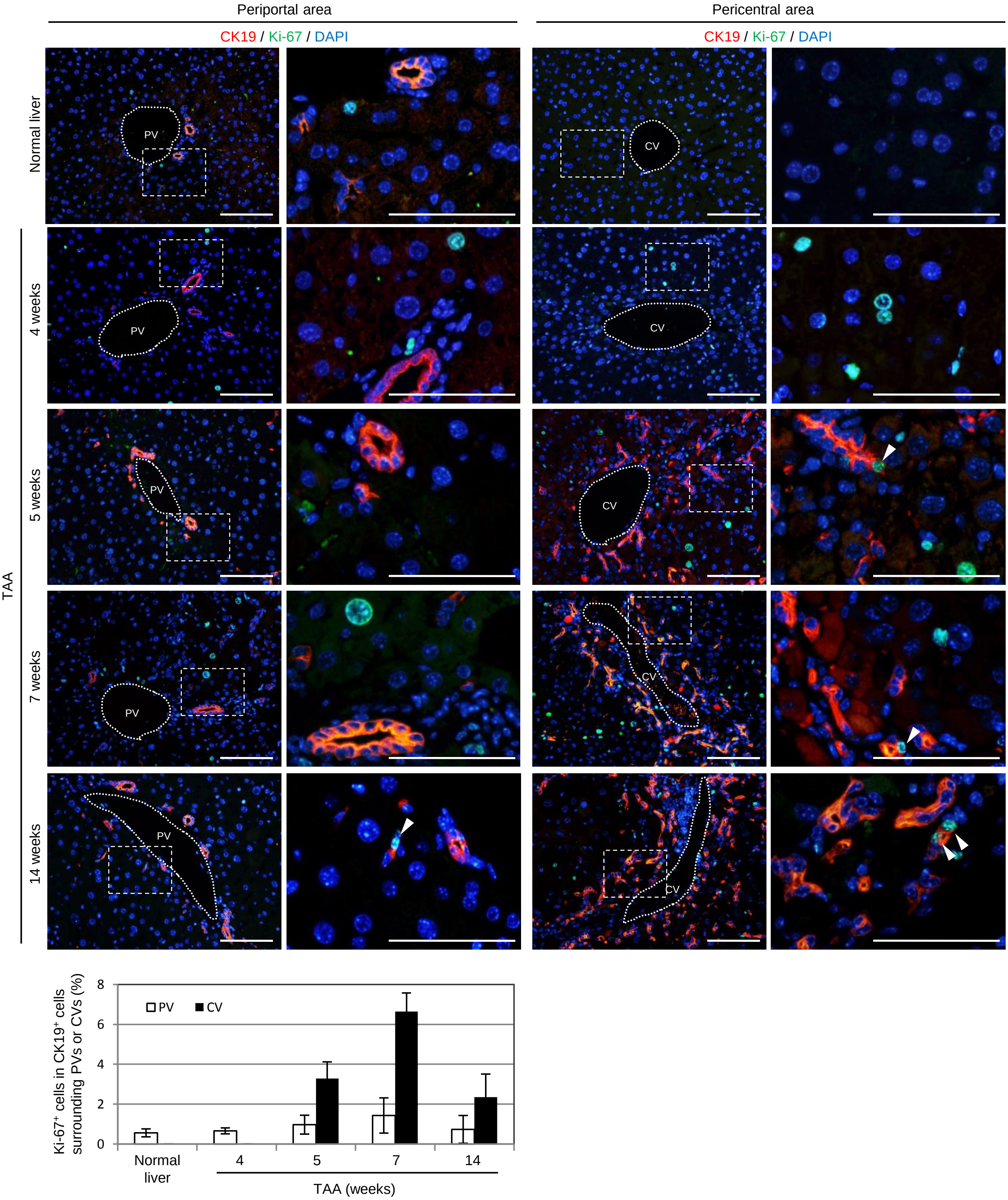

Terada et al., Supplementary Figure 2

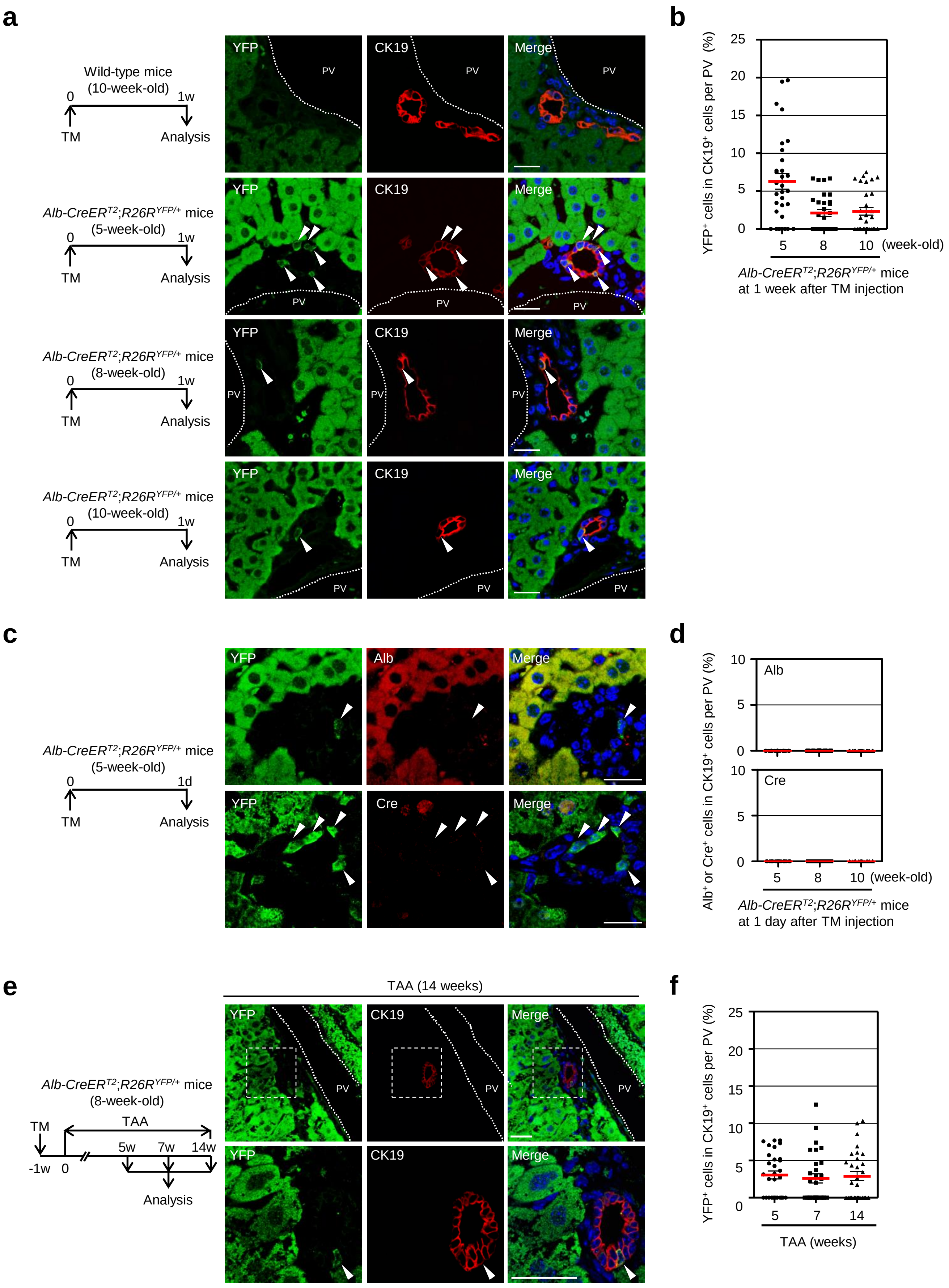

Terada et al., Supplementary Figure 3

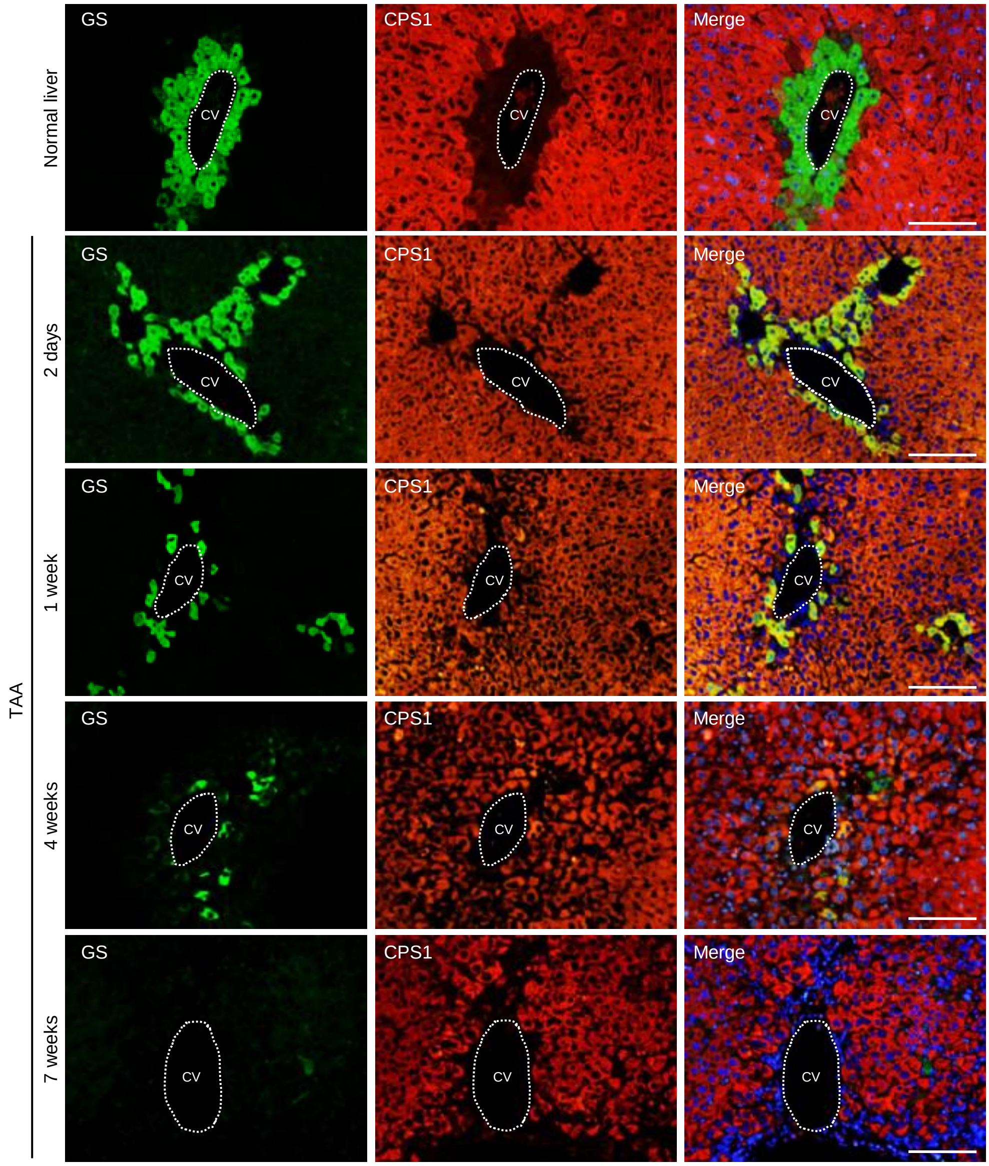

Terada et al., Supplementary Figure 4

a

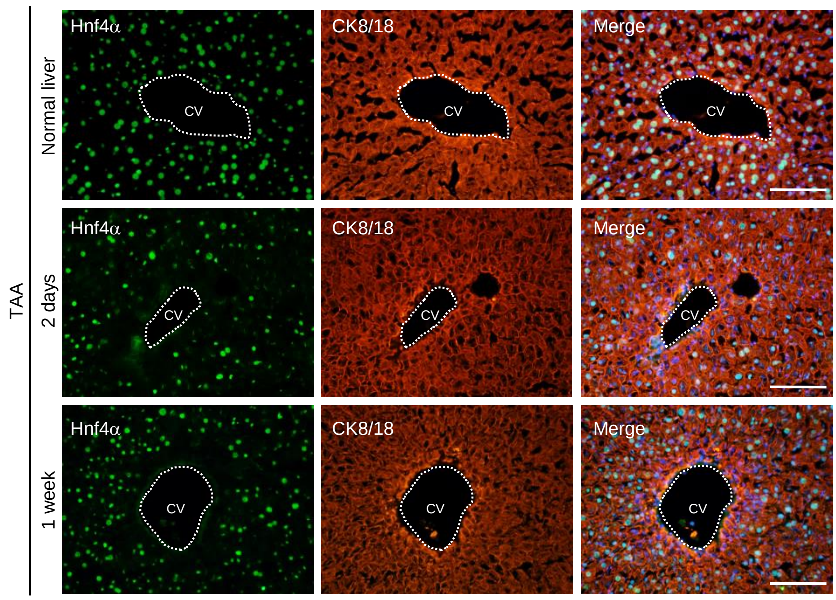

b

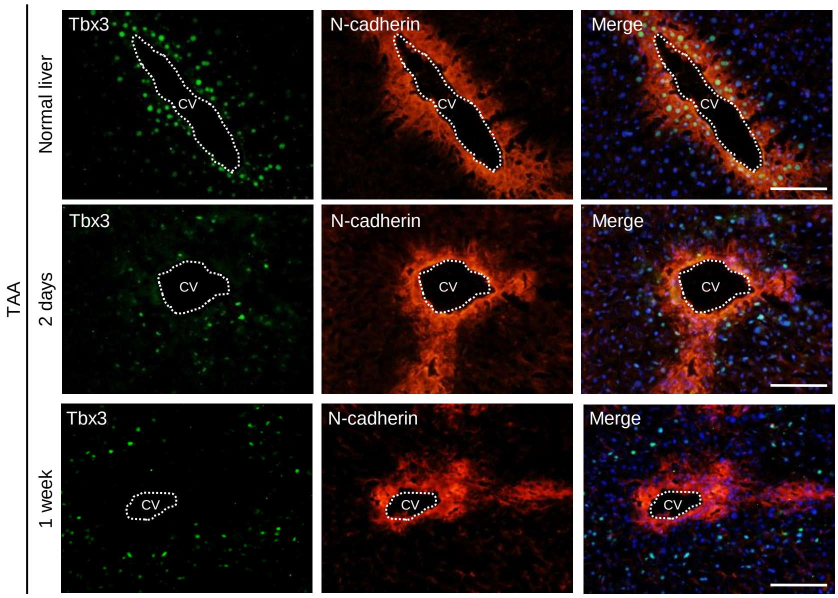

Terada et al., Supplementary Figure 5

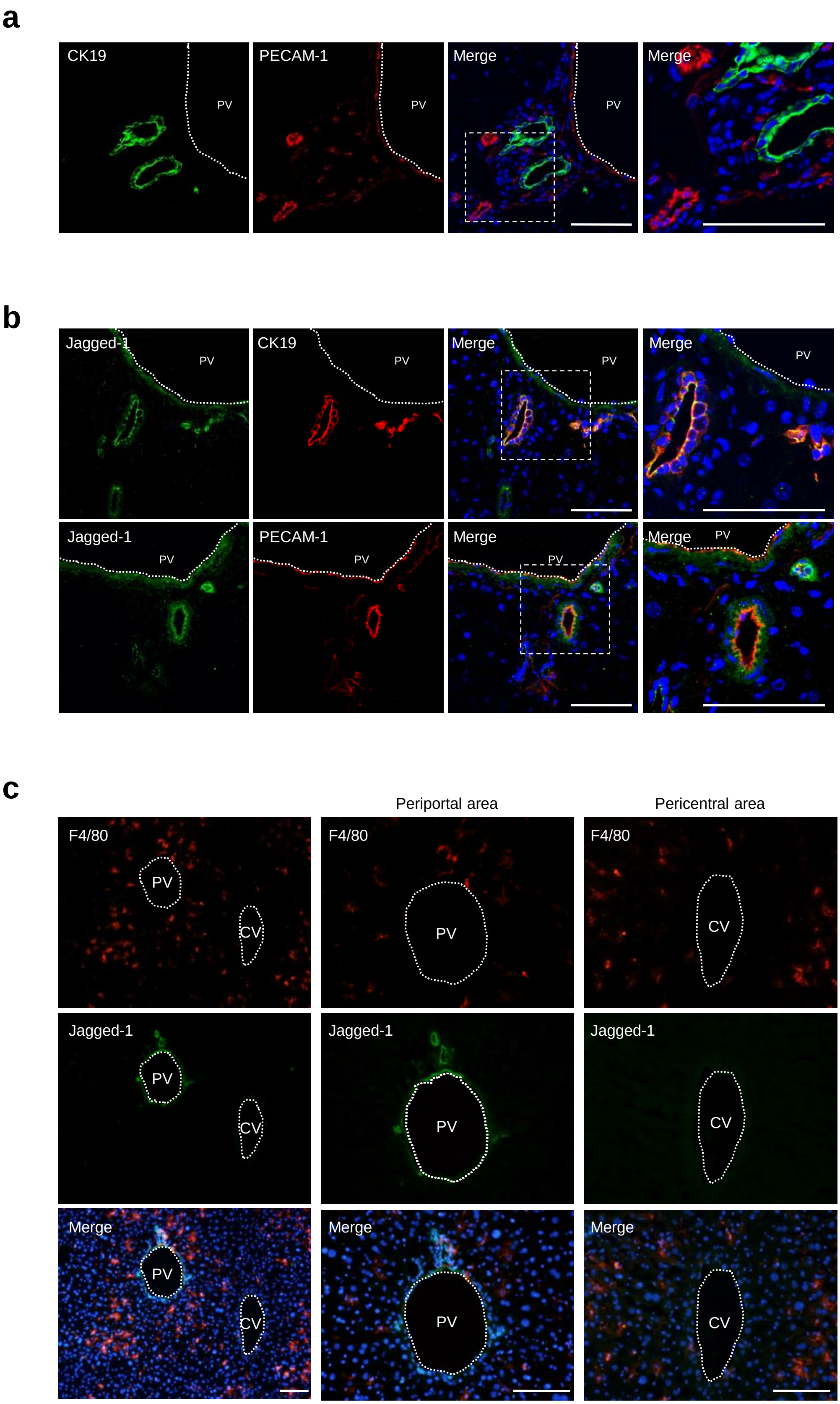

Terada et al., Supplementary Figure 6

a

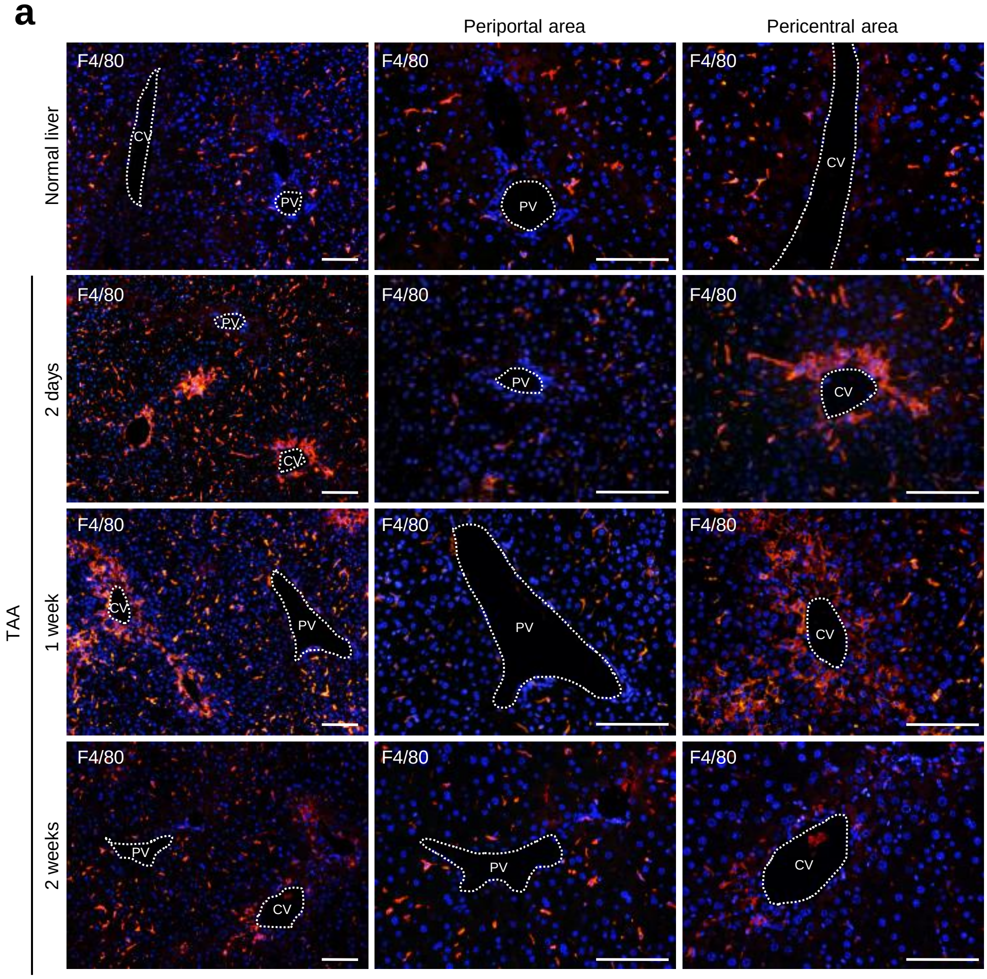

b

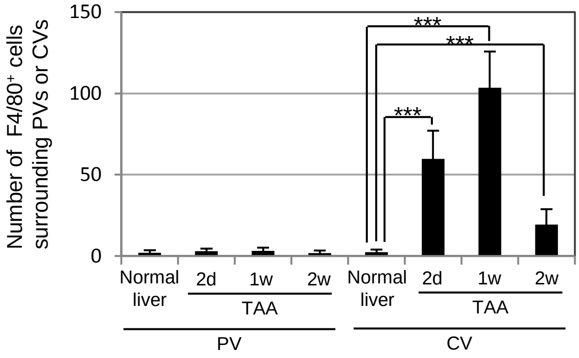

Terada et al., Supplementary Figure 7

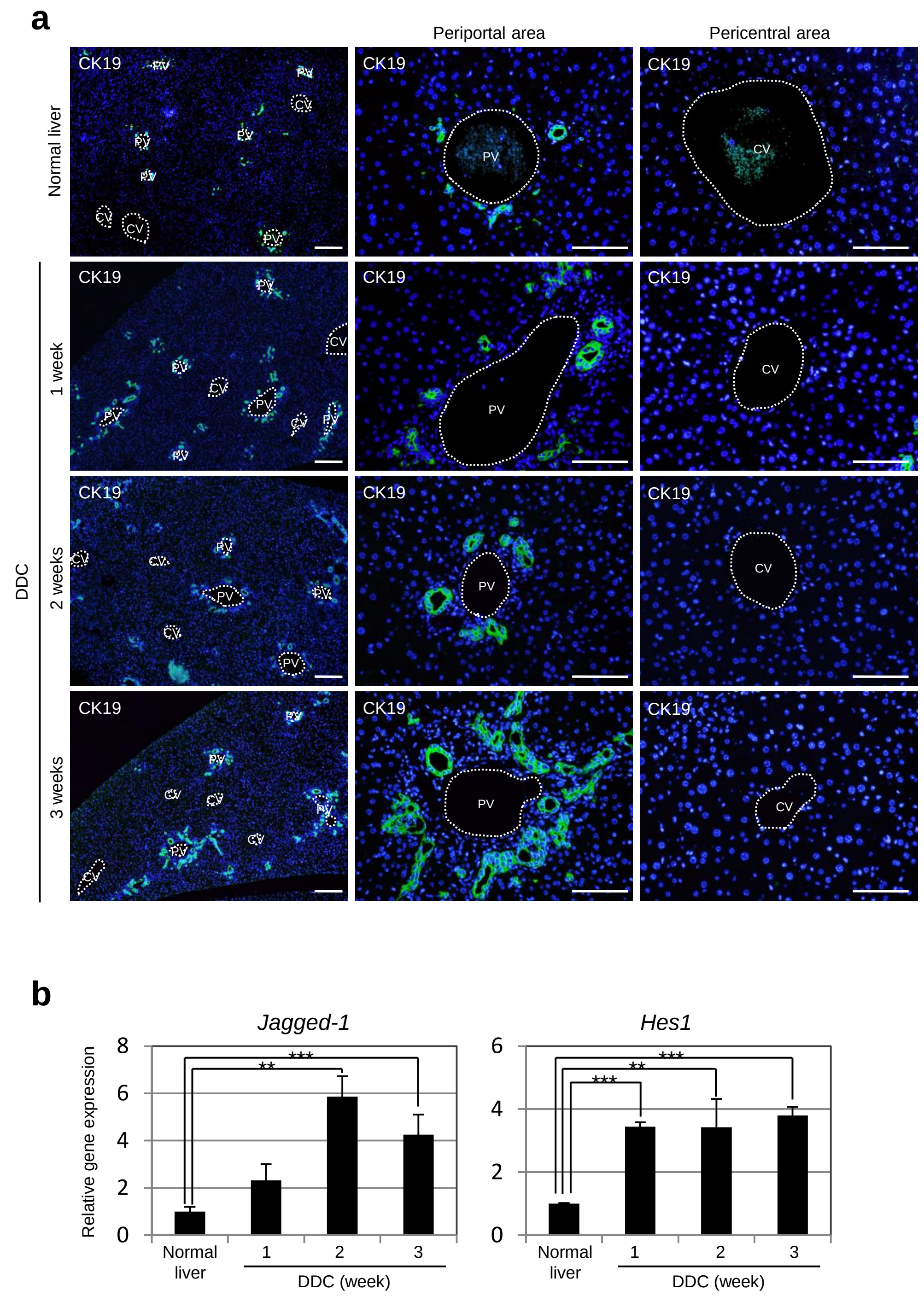

Terada et al., Supplementary Figure 8

a

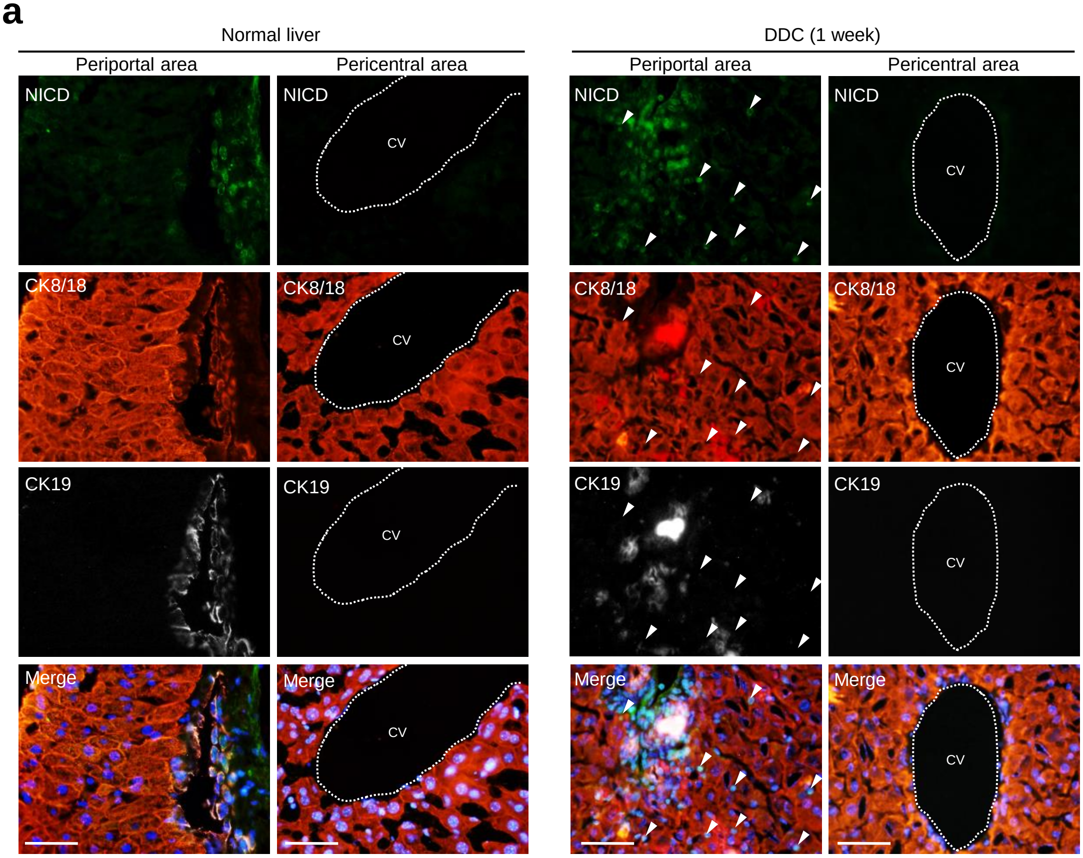

b

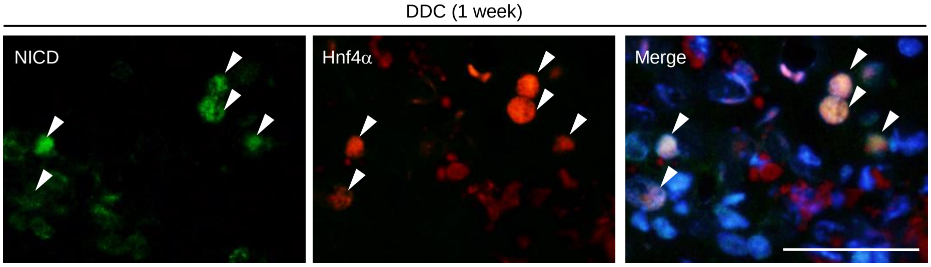

Terada et al., Supplementary Figure 9

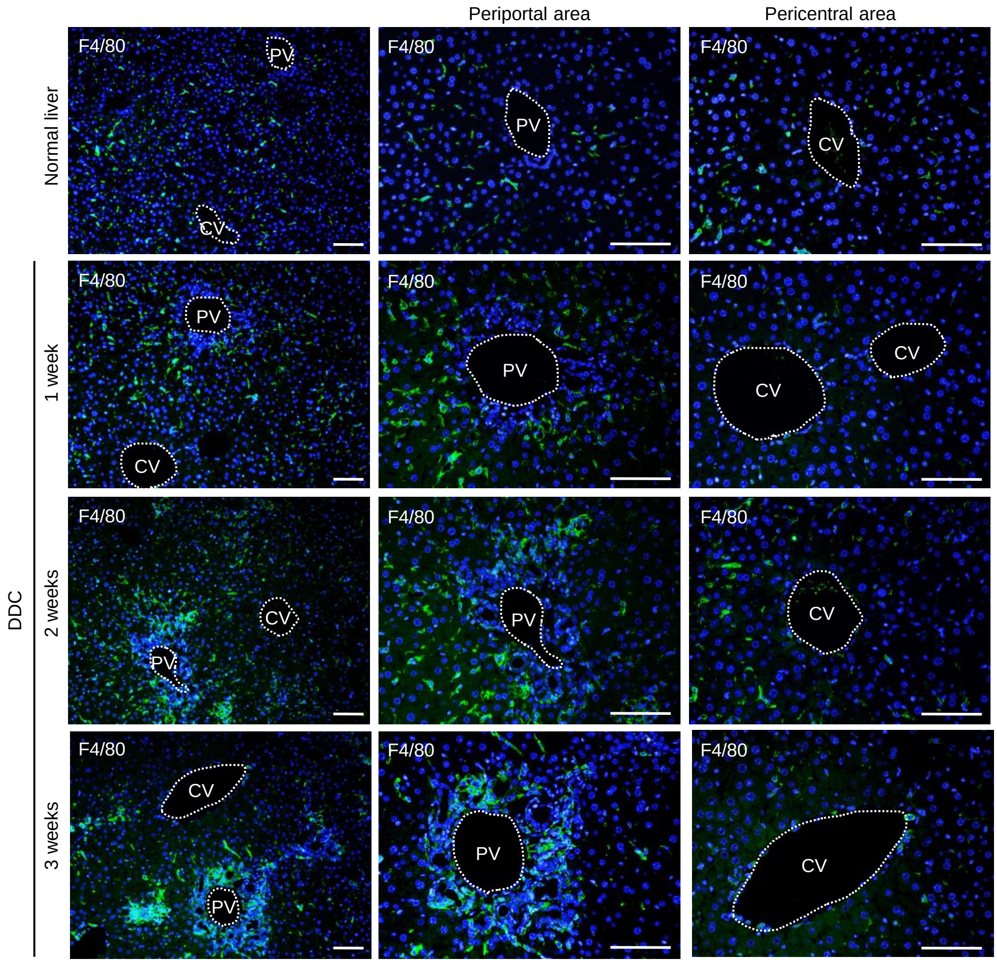

Terada et al., Supplementary Figure 10

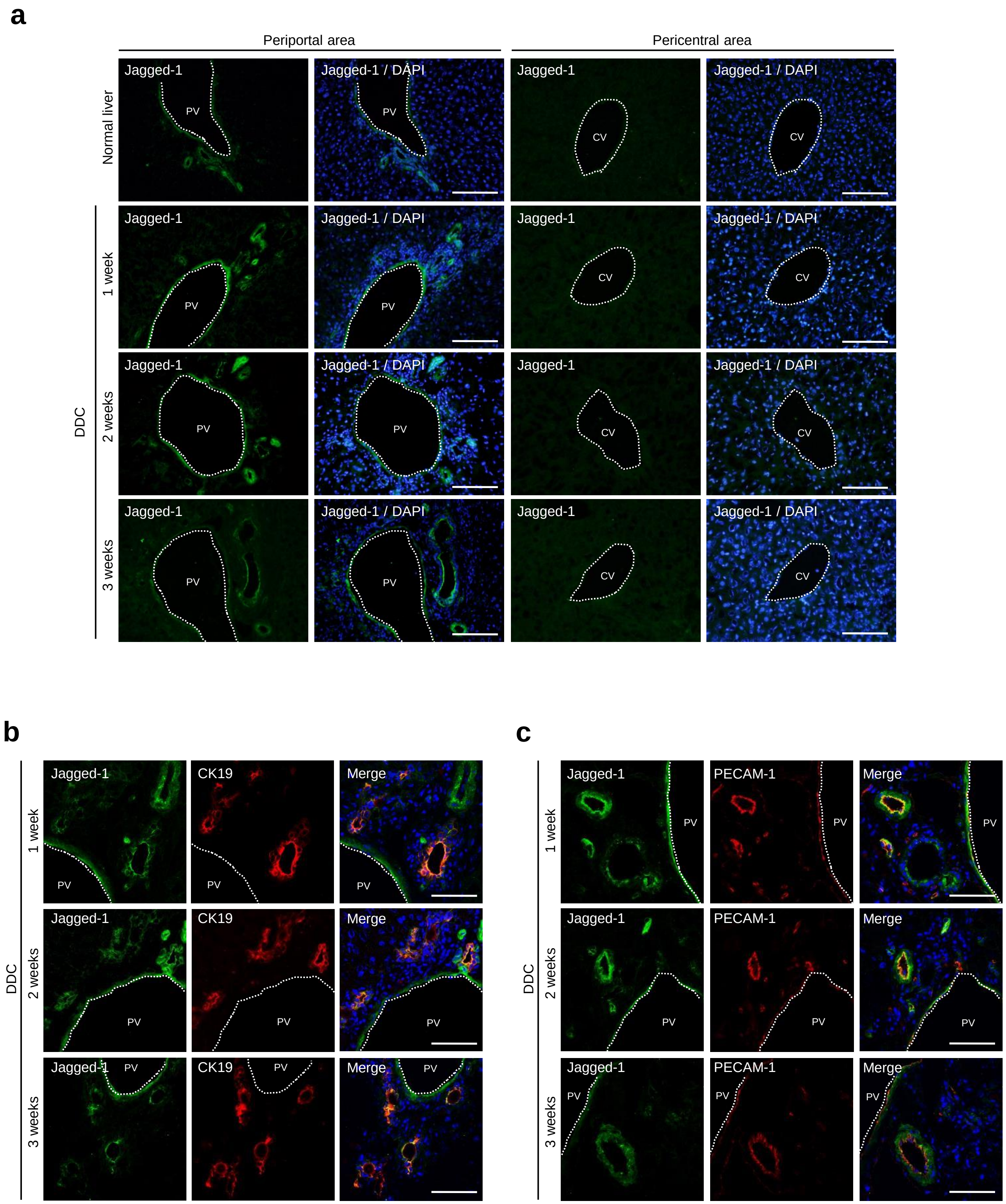

Terada et al., Supplementary Figure 11

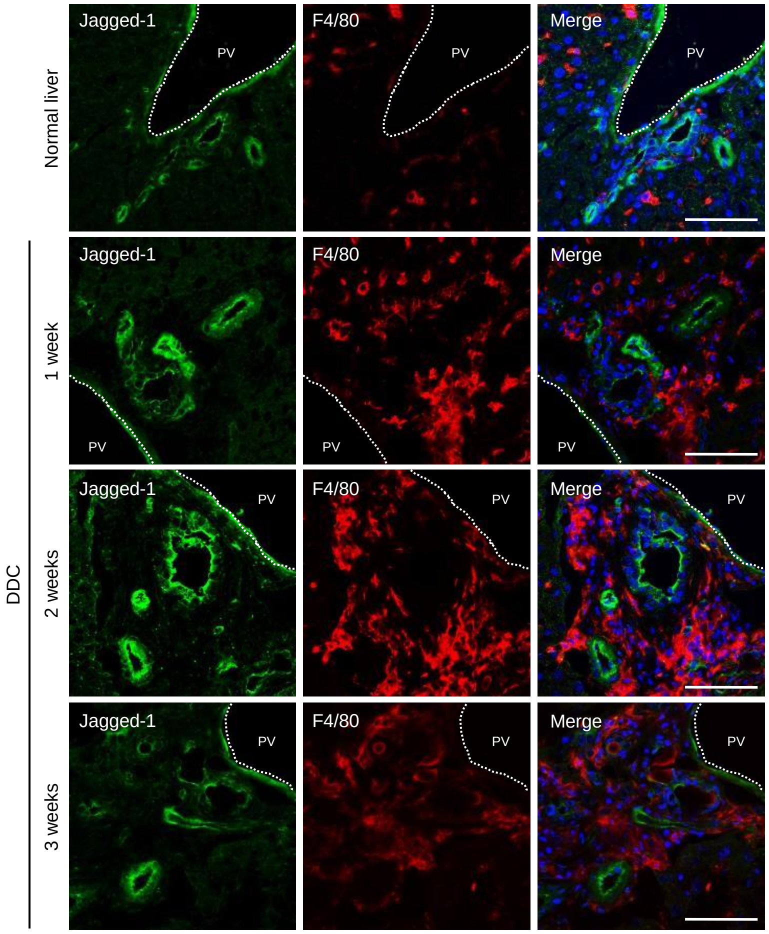

Terada et al., Supplementary Figure 12

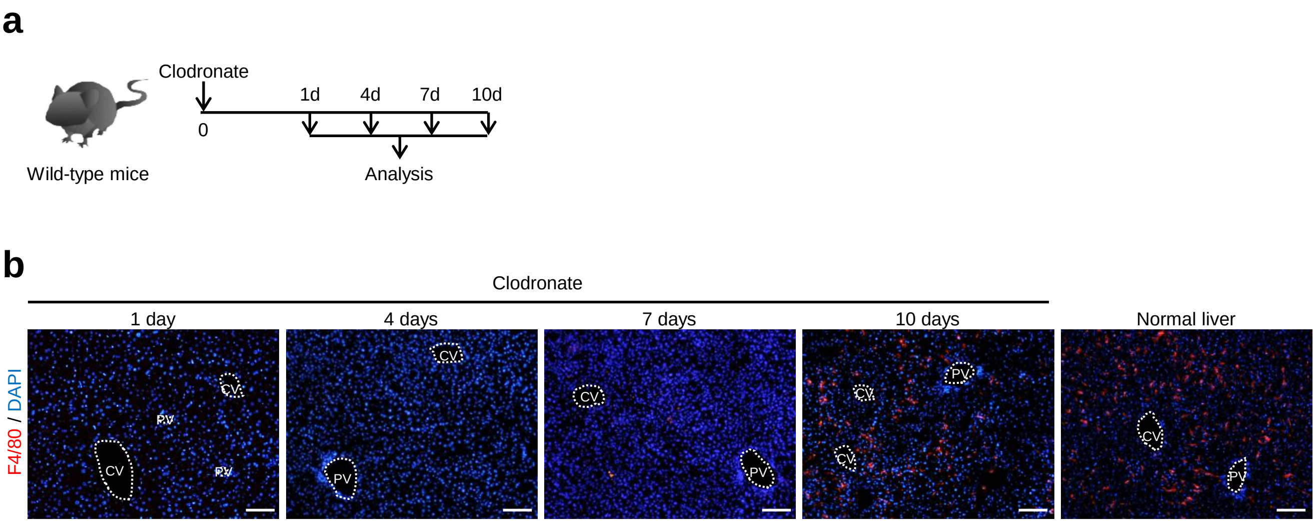

Terada et al., Supplementary Figure 13

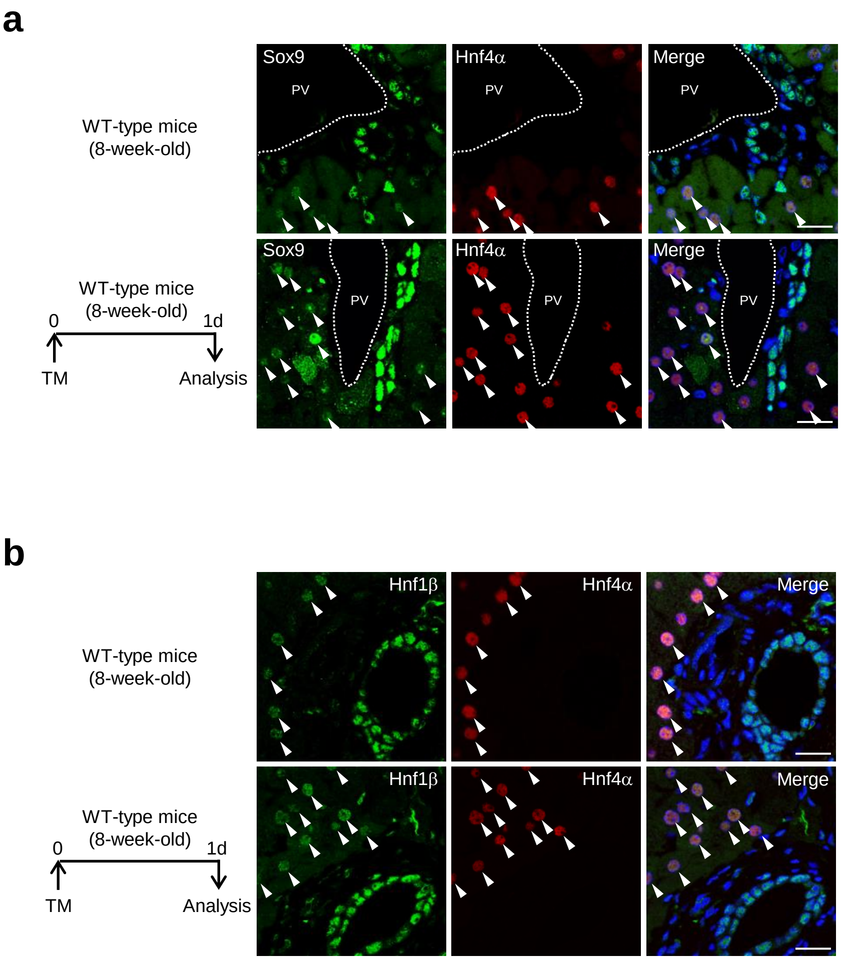

Terada et al., Supplementary Figure 14

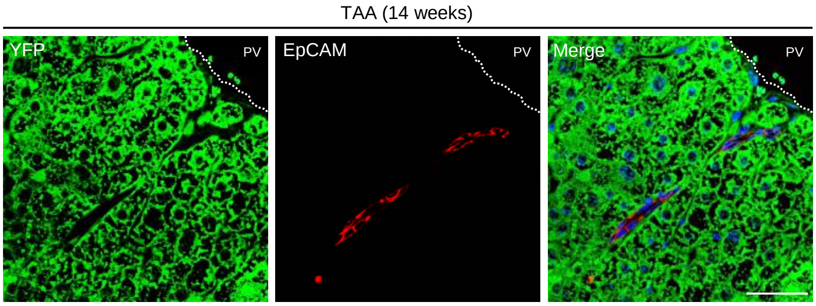

Terada et al., Supplementary Table 1

Primary antibody	Species	Source	Catalog number	Dilution
CK19	Rabbit	Made in previous study	Sekiya, S. & Suzuki, A. *J. Clin. Invest.* **122,** 3914-3918 (2012).	1:2000
CK19	Rat	Made in this study	-	1:100
EpCAM	Rabbit	Abcam	ab71916	1:500
OPN	Goat	R&D Systems	AF808	1:500
GFP / YFP	Goat	Abcam	ab6673	1:2000
Ki67	Rabbit	Abcam	ab833-500	1:500
Alb	Mouse	Sigma	A6684	1:400
Cre	Rabbit	Covance	PRB106P	1:500
Jagged-1	Rabbit	Santa Cruz	sc-8303	1:50
NICD	Rabbit	Cell signaling	#4147	1:100
α-SMA	Mouse	Abcam	A2547	1:500
PECAM-1	Rat	BD Pharmingen	550274	1:200
CK8/18	Guinia Pig	PROGEN Biotechnik	GP11	1:500
Thy1.2	Rat	BD Pharmingen	140301	1:200
F4/80	Rat	Bio Rad	MCA497G	1:500
Hnf4α	Mouse	Perseus Proteomics	PP-K9218-00	1:500
Tbx3	Goat	Santa Cruz	sc-17871	1:500
N-cadherin	Mouse	BD Pharmingen	610920	1:500
Hnf1β	Rabbit	Santa Cruz	sc-22840	1:500
Sox9	Rabbit	Millipore	AB5535	1:500
GS	Mouse	Abcam	ab64613	1:1000
Cleaved caspase-3	Rabbit	Cell signaling	#9661	1:500
CPS1	Rabbit	Abcam	ab129076	1:1000
